# Tumor immunotherapies by immune checkpoint inhibitors (ICIs); the pros and cons

**DOI:** 10.1186/s12964-022-00854-y

**Published:** 2022-04-07

**Authors:** Adel Naimi, Rebar N. Mohammed, Ahmed Raji, Supat Chupradit, Alexei Valerievich Yumashev, Wanich Suksatan, Mohammed Nader Shalaby, Lakshmi Thangavelu, Siavash Kamrava, Navid Shomali, Armin D. Sohrabi, Ali Adili, Ali Noroozi-Aghideh, Ehsan Razeghian

**Affiliations:** 1 Cellular and Molecular Research Center, Sabzevar University of Medical Sciences, Sabzevar, Iran; 2 Medical Laboratory Analysis Department, Cihan University Sulaimaniya, Sulaymaniyah, 46001 Kurdistan Region Iraq; 3 College of Veterinary Medicine, University of Sulaimani, Suleimanyah, Iraq; 4 College of Medicine, University of Babylon, Department of Pathology, Babylon, Iraq; 5 Department of Occupational Therapy, Faculty of Associated Medical Sciences, Chiang Mai University, Chiang Mai, 50200 Thailand; 6 Department of Prosthetic Dentistry, Sechenov First Moscow State Medical University, Moscow, Russia; 7 Faculty of Nursing, HRH Princess Chulabhorn College of Medical Science, Chulabhorn Royal Academy, Bangkok, 10210 Thailand; 8 Associate Professor of Biological Sciences and Sports Health Department, Faculty of Physical Education, Suez Canal University, Ismailia, Egypt; 9 Department of Pharmacology, Saveetha Dental College, Saveetha Institute of Medical and Technical Science, Saveetha University, Chennai, India; 10 Department of Surgery, School of Medicine, Shahid Beheshti University of Medical Sciences, Tehran, Iran; 11 Immunology Research Center (IRC), Tabriz University of Medical Sciences, Tabriz, Iran; 12 Student Research Committee, Tabriz University of Medical Sciences, Tabriz, Iran; 13 Department of Oncology, Tabriz University of Medical Sciences, Tabriz, Iran; 14 Department of Hematology, Faculty of Paramedicine, Tehran University of Medical Sciences, Tehran, Iran; 15 Human Genetics Division, Medical Biotechnology Department, National Institute of Genetics Engineering and Biotechnology (NIGEB), Tehran, Iran

**Keywords:** Immune checkpoint inhibitors, CTLA-4, PD-1/PD-L1, Cancer, Immunotherapy

## Abstract

The main breakthrough in tumor immunotherapy was the discovery of immune checkpoint (IC) proteins, which act as a potent suppressor of the immune system by a myriad of mechanisms. After that, scientists focused on the immune checkpoint molecules mainly. Thereby, much effort was spent to progress novel strategies for suppressing these inhibitory axes, resulting in the evolution of immune checkpoint inhibitors (ICIs). Then, ICIs have become a promising approach and shaped a paradigm shift in tumor immunotherapies. CTLA-4 plays an influential role in attenuation of the induction of naïve and memory T cells by engagement with its responding ligands like B7-1 (CD80) and B7-2 (CD86). Besides, PD-1 is predominantly implicated in adjusting T cell function in peripheral tissues through its interaction with programmed death-ligand 1 (PD-L1) and PD-L2. Given their suppressive effects on anti-tumor immunity, it has firmly been documented that ICIs based therapies can be practical and rational therapeutic approaches to treat cancer patients. Nonetheless, tumor inherent or acquired resistance to ICI and some treatment-related toxicities restrict their application in the clinic. The current review will deliver a comprehensive overview of the ICI application to treat human tumors alone or in combination with other modalities to support more desired outcomes and lower toxicities in cancer patients.

**Video Abstract**

**Supplementary Information:**

The online version contains supplementary material available at 10.1186/s12964-022-00854-y.

## Background

The promising rise and achievement of cancer immunotherapy over the past decade has developed the clinical management of many malignancies that were beforehand endowed with poor prognosis [1, 2]. Immune-checkpoint inhibitors (ICIs) are the leading approaches in tumor immunotherapy. They have been considered in the treatment of tumors due to their comprehensive bioactivity in various histological tumors, the stability of their response, and therapies that are evident even in metastatic and chemotherapy-resistant malignancies [3]. Interaction between immune checkpoints and their ligands negatively modifies T cell function and responding pathways complicated in the physiological immune response against tumor-associated antigens (TAAs). The immune checkpoints and their responding ligands are commonly upregulated in the TME of many human malignancies, and they signify substantial barricades for initiation of effective anti-tumor immune reaction [4, 5].

**Fig. 1:**
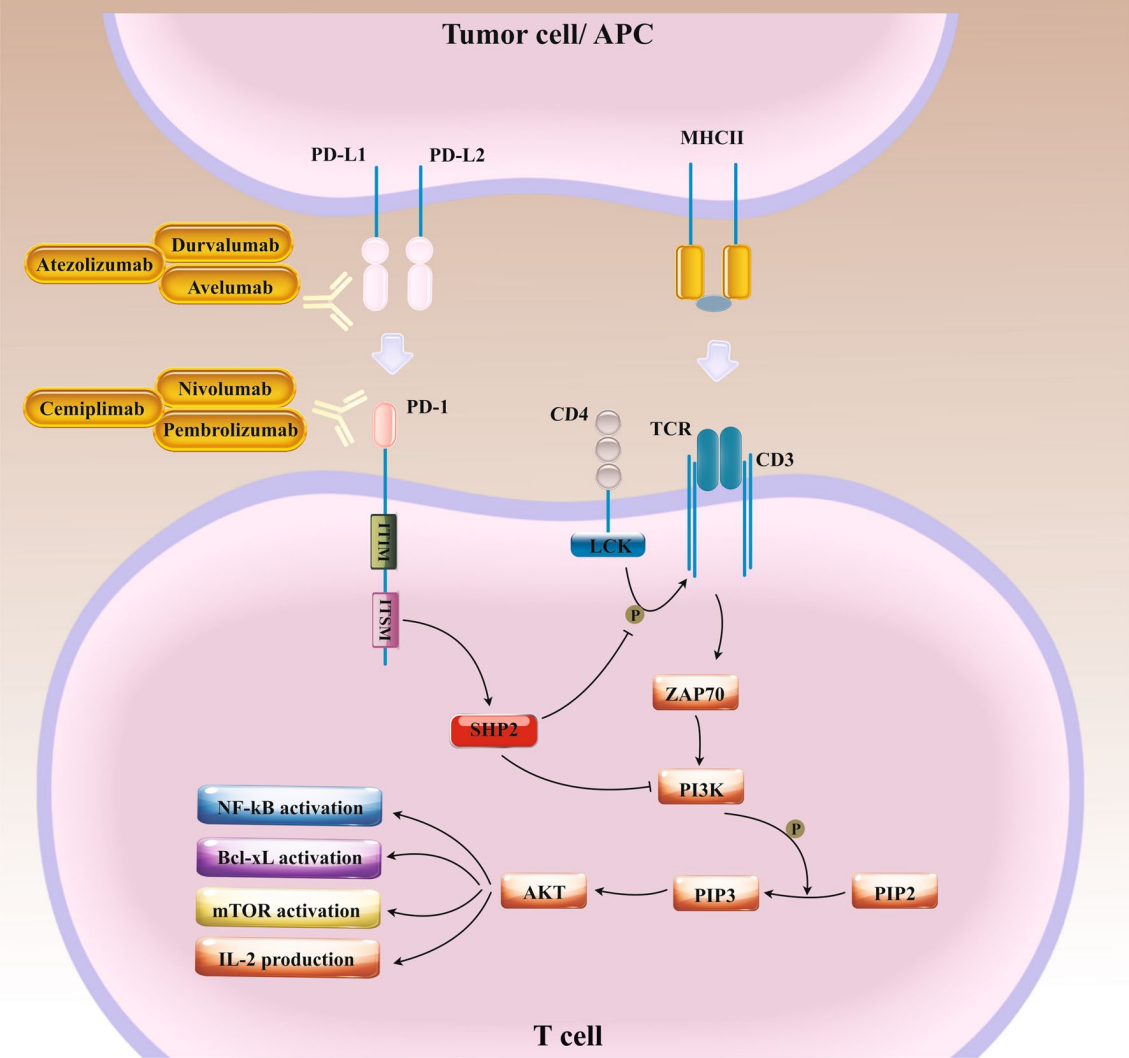
The inhibitory effects of the CTLA-4/B7 on T cell anti-tumor activities. CTLA-4 is expressed on activated T cells, is about 30% homologous with CD28 and binds to the same ligands as CD28, known as B7-1 and B7-2 expressed on APCs or tumor cells. This interaction results in activation of SHP2 and so down-regulation of PI3K/AKT axis. Cytotoxic T-lymphocyte-associated protein 4 (CTLA-4), SH2 containing protein tyrosine phosphatase-2 (SHP2), Phosphoinositide 3-kinases (PI3Ks), Phosphatidylinositol-4,5-bisphosphate (PIP2), Phosphatidylinositol (3,4,5)-trisphosphate (PIP3), Lymphocyte-specific protein tyrosine kinase (LCK), T cell receptor (TCR), Nuclear factor-κB (NF-κB), Mammalian target of rapamycin (mTOR), B-cell lymphoma-extra large (Bcl-xL), Major histocompatibility complex class II (MHCII), Interleukin-2 (IL-2)

**Fig. 2:**
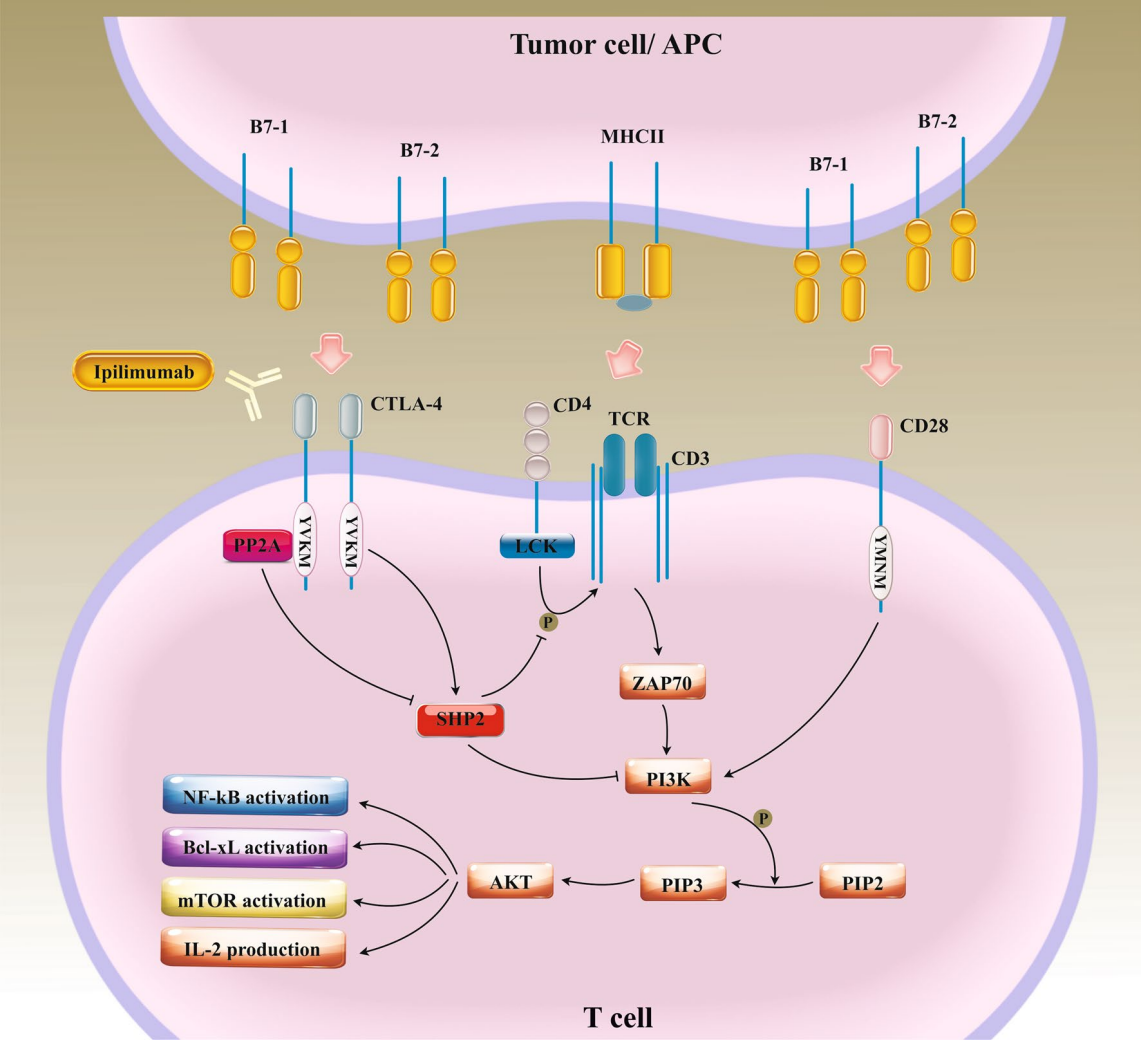
The inhibitory effects of the PD-1/PD-L interactions on T cell anti-tumor activities. PD-L1 expressed on APCs or tumor cells following interaction with PD-1 dysregulated on the surface of activated T cell limits self-reactive T cell proliferation and cytokine production as a result of activation of SHP2, which down-regulates PI3K/AKT axis. Programmed cell death protein 1(PD-1), Programmed death-ligand 1 and 2 (PD-L1, PD-L2), Antigen-presenting cells (APCs), SH2 containing protein tyrosine phosphatase-2 (SHP2), Phosphoinositide 3-kinases (PI3Ks), Phosphatidylinositol-4,5-bisphosphate (PIP2), Phosphatidylinositol (3,4,5)-trisphosphate (PIP3), Lymphocyte-specific protein tyrosine kinase (LCK), T cell receptor (TCR), Nuclear factor-κB (NF-κB), Mammalian target of rapamycin (mTOR), B-cell lymphoma-extra large (Bcl-xL), Major histocompatibility complex class II (MHCII), Interleukin-2 (IL-2)

Among the checkpoint-blocking approaches, the two most eminent are blocking cytotoxic-T-lymphocyte-associated protein 4 (CTLA-4 or CD152) and targeting the interaction between programmed cell death 1 (PD-1 or CD279) and programmed cell death ligand 1 (PD-L1 or CD274 or B7 homolog 1) [6]. Co-stimulation of CD80/CD86 via CD28 provides essential stimulus signals that support T cell proliferation and effective differentiation throughout the induction phase of the immunological response [7]. Structurally, the CTLA-4 has substantial homology to the costimulatory molecule CD28. It can also bind B7 molecules on antigen-presenting cells (APCs) with much higher affinity and avidity than CD28 [8]. The CTLA-4 co-inhibitory receptor is found on lately induced T cells and interacts with the identical ligands as CD28 but with higher affinity [9, 10]. It has a unique YVKM motif at the cytoplasmic domain, which binds to the SHP-2 and elicits inhibitory signaling like PD-1 (Fig. 1) [11]. Conversely, Src homology two domain-containing protein tyrosine phosphatase 2 (SHP-2) inhibitors typically provoke anti-tumor immunity, such as enhancing T cell cytotoxic activities and immune-mediated tumor regression [12]. CTLA-4 on T cells throughout the induction phase of an anti-cancer immune reaction obstructs T cell activation by inhibiting the formation of interaction between CD80/CD86 and CD28 and conveying inhibitory signals, directly suppressing T cell activation [13, 14]. The information respecting the CTLA-4 activities has led to the theory that hindering its action could ease T cell responses to persist, which has implications for progressing an understanding of tumor immunology around that time [11]. Many preclinical proofs have sustained this theory, inspiring the manufacture of ipilimumab, a monoclonal antibody (mAb) against human CTLA-4, to use in the clinic [15]. CTLA-4 blocker therapy, despite documented activities in concomitant activation, can also deplete regulatory T cells (Treg) from the tumor microenvironment (TME) as a result of high CTLA-4 expression at the Treg level [16]. As loss of CTLA-4 may perturb immunosuppressive effects of Treg on tumor-infiltrating lymphocytes (TILs) [17], it has been robustly proved that CTLA-4 blockade can support a paradigm shift in tumor therapy. Besides, the activity of PD-1 as an immune checkpoint was recognized following the detection of one of its ligands, PD-L1 [18]. Like CTLA-4, the PD-1is expressed on activated T cells, and its functions have been found to abolish signaling mediated on antigen recognition by the T cell receptor [19]. PD-1 has two ligands, including PD-L1 and PD-L2. While PD-L2 is mainly expressed on APCs, PD-L1 can be found on various cell types, comprising tumor cells, immune cells, epithelial cells, and endothelial cells [20]. The cytoplasmic tail of PD-1 shall consist of two tyrosine-based structural motifs, an immunoreceptor tyrosine-based inhibitory motif (ITIM) (V/L/I/XpYXX/L/V) and an immunoreceptor tyrosine-based switch motif (ITSM) (TXpYXXV/I) (Fig. 2) [21]. There is clear evidence showing that PD-1 inhibitory function relies on the ITSM phosphotyrosine, which in turn recruits SHP2 and consequently suppresses downstream signaling axes [21, 22]. PD-L1 expression has been allied with exposure to interferon-γ (IFN-γ), for instance upon anti-tumor T helper type 1 (Th1) cell responses. It leads potently to tumor cells evasion from T cell immunosurveillance [23–25]. Indeed, the activities of PD-1 in immune cells comprise the stimulation of the maintenance of peripheral immune tolerance, defending tissue from immune attack, and diminishing infectious immunity and also tumor immunity [20]. Like anti-CTLA-4 antibody ipilimumab, PD-1/PD-L1 inhibitors nivolumab, pembrolizumab, cemiplimab, atezolizumab, avelumab, and durvalumab have received FDA approvals since 2011 [26]. Notwithstanding, tumor resistance to ICIs [27, 28] along with the ICIs mediated toxicities [29] hinder their clinical application. For instance, the response rate for melanoma patients treated with pembrolizumab (anti-PD-1) was only 33%, and also about 20–30% of patients with lung carcinoma mainly experienced desired outcomes upon ICIs blockade therapy [30].

Here, we've focused on the therapeutic application of ICIs as a pioneering approach in the field of tumor immunotherapy. Moreover, recent findings on combination therapy with ICIs to defeat tumor resistance against them will be stated.

## Association between immune checkpoints (IC) proteins and tumor prognosis

To date, the predictive value of the ICs has been documented in a diversity of human malignancies. A study in 773 patients with stage I-III colorectal cancer (CRC) patients indicated that high tumoral immune checkpoints (lymphocyte-activation gene 3) LAG-3 and PD-1 were associated with poor survival. In contrast, patients with high T-cell immunoglobulin and mucin-domain containing-3 (TIM-3), LAG-3, and PD-1 on immune cells within the stroma showed better survival [31]. Another investigation on 398 tumor tissues from stage I to IV gastric cancer patients revealed that higher tumor-infiltrating lymphocyte (TIL) frequency was associated with a lower risk of tumor development and showed survival advantages in gastric cancer patients [32]. Also, PD-L1 directly displayed a significant correlation with high TIL infiltration and tumor regression [32]. These findings signified that induction of immune checkpoint within gastric cancer patients could bring about a high immune infiltration frequency, directing patient selection for checkpoint blockade therapy [32]. The high densities of TILs, high ratios of PD-1+/CD8+ cells, and high levels of PD-L1 have been suggested that are negatively associated with melanoma brain metastases size, and high levels of PD-L1 may support a marked trend towards better survival [33]. However, there are some other inconsistent reports. For instance, studies in NSCLC patients treated with concurrent chemoradiotherapy (CRT) revealed that PD-L1 expression < 1% on tumor cells was related to improved overall survival (OS), and also patients with low CD8+ TIL density exhibited better OS [34]. Gennen et al. found that longest and shortest OS were attained in patients with type I (PD-L1neg/CD8low) and type IV (PD-L1pos/CD8low) tumors, respectively [34]. Similarly, evaluation of CD8+ T cell infiltration and expression of immune checkpoints, such as PD-1, PD-L1, PD-L2, and T cell immunoreceptor with Ig and ITIM domains (TIGIT) in 154 patients with primary esophageal squamous cells carcinoma (ESCCs) suggested that the number of PD-1+ TILs was positively correlated with CD8+ TILs [35]. The analysis demonstrated that enhanced quantities of PD-1+ and TIGIT+ TILs and PD-L1 and PD-L2 expression were related to a shorter OS [35]. Other studies in this regard also revealed that PD-1, PD-L1, TIM-3, LAG-3 immune checkpoints were positively associated with epithelial-mesenchymal transition (EMT) status in ESCC [36], and also tumor progress in breast cancer [37]. In metastatic breast tumors, PDL-L1high circulating tumor cells (CTC) were accompanied with the disease progression and shorter progression-free survival (PFS) [38]. PD-L1 has independent worse prognostic implications in metastatic breast cancer, indicating a possible role of innate and adaptive immune escape mechanisms in breast cancer metastatic potential [38]. Other studies in 135 primary clear cell renal cell carcinoma (ccRCC) tumors have also represented that high expression of ICs in the lack of fully functional mature dendritic cells (DCs) was in association with promoted risk of disease progression [39]. In contrast, low expression of ICs and also localization of mature DC in peritumoral immune aggregates might indicate desisted prognosis [39]. Indeed, expression of ICs expression and DC localization in the TME seems to hinder the clinical activities of CD8+ T cells in ccRCC [39].

These inconsistent reports have outlined the significance of patient selection based on tumor microenvironment characteristics for checkpoint blockade therapy.

## The U.S. Food and Drug Administration (FDA)-approved ICIs

Tumor cells can trigger diverse ICs pathways to harbor immunosuppressive functions. Recent developments in our knowledge respecting T cell immunobiology have been chiefly involved in designing therapeutic strategies or molecules to circumvent tumor immune evasion mechanisms. Meanwhile, monoclonal antibodies (mAb) targeting ICs have delivered a massive breakthrough in tumor therapeutics [40–42]. Among the ICIs, PD-1/PD-L1 and CTLA-4 inhibitors have exhibited encouraging therapeutic outcomes, and some have been indicated for various tumor treatments since 2011 (Table 1), whereas others are under clinical trials [26]. In the context of cancer, where adverse T cell regulatory pathways are often overactive, immune checkpoint blockade has proven to be an effective strategy for enhancing the effector activity and clinical impact of anti-tumor T cells [26]. These ICIs have persuaded desired and durable responses in a significant proportion of cancer patients.

**Table 1:** Current FDA-approved immune checkpoint inhibitors (ICIs)

Agent	Target IC	Approved conditions
Ipilimumab	CTLA-4	Melanoma, MSI-H/dMMR colorectal cancer (CRC), renal cell carcinoma (RCC) (in combination with nivolumab)
Nivolumab	PD-1	MSI-H or dMMR colorectal cancer (CRC), head and neck squamous cell carcinomas (HNSCC), hepatocellular carcinoma (HCC), melanoma, Classic Hodgkin lymphoma (cHL), non-small-cell lung carcinoma (NSCLC), renal cell carcinoma (RCC), urothelial cancer, small-cell lung carcinoma (c-SCLC)
Pembrolizumab	PD-1	Cervical cancer, gastric cancer, head and neck squamous cell carcinomas (HNSCC), hepatocellular carcinoma (HCC), Classic Hodgkin lymphoma (cHL), melanoma, Merkel cell carcinoma (MCC), MSI-H or dMMR colorectal cancer (CRC), non-small-cell lung carcinoma (NSCLC), diffuse large B-cell lymphoma (DLBCL), urothelial cancer
Cemiplimab	PD-1	Cutaneous squamous cell carcinoma (cSCC)
Atezolizumab	PD-L1	Non-small-cell lung carcinoma (NSCLC), urothelial cancer
Avelumab	PD-L1	Merkel cell carcinoma (MCC), urothelial cancer
Durvalumab	PD-L1	Non-small-cell lung carcinoma (NSCLC), urothelial cancer

Programmed cell death protein 1 (PD-1), programmed death-ligand 1 (PD-L1), cytotoxic-T-lymphocyte-associated protein 4 (CTLA-4), microsatellite instability-high (MSI-H)/mismatch repair deficient (dMMR)

### CTLA-4 inhibitors

Ipilimumab is a fully human IgG1 monoclonal antibody (mAb) targeting CTLA-4; it was the first FDA-approved ICI in 2011 for patients suffering from advanced melanoma [43]. Ipilimumab avoids T-cell suppression and stimulates the effector T cell’s activation and proliferation. This antibody, in combination with nivolumab as the PD-1 inhibitor, has been approved for the treatment of patients with metastatic colorectal cancer (CRC) with microsatellite instability-high (H-MSI) or mismatch repair (MMR) deficiencies [44]. Besides, its application along with nivolumab has been approved in patients with intermediate- or poor-risk renal cell carcinoma (RCC) regardless of PD-L1 status [45], as well as in patients with hepatocellular carcinoma (HCC) who have previously been treated with sorafenib [46]. In addition, ipilimumab plus nivolumab has also been indicated for the first-line treatment of patients with non-small-cell lung carcinoma (NSCLC) whose tumors express PD-L1 (≥ 1%) [47], and also for malignant pleural mesothelioma (MPM) [48].

### PD-1 inhibitors

In addition to the nivolumab, which is a fully human IgG4 mAb, two other PD-1 inhibitors, pembrolizumab (IgG4 mAb) and cemiplimab (IgG4 mAb), have demonstrated promising outcomes in melanoma and NSCLC patients [49]. Interfaces between PD-1 and its ligands, B7-H1/PD-L1 and B7-DC/PD-L2, ultimately lead to T cells' inactivation to support immune homeostasis and prevent autoimmunity [49]. Apart from the combination therapy, monotherapy with nivolumab is also the first FDA-approved immunotherapy for gastric cancer's first-line treatment and is an effective treatment for NSCLC, classic Hodgkin's lymphoma (cHL), and melanoma [50]. Since 2019, pembrolizumab has been approved for the treatment of patients with metastatic melanoma, metastatic NSCLC in certain situations [51], as a first-line treatment for metastatic bladder cancer [52], as a second-line treatment for head and neck squamous cell carcinomas (HNSCC) [53], and also for refractory cHL [54], and metastatic ESCC [55]. Besides, cemiplimab was approved to treat basal cell carcinoma, cutaneous squamous cell carcinoma (CSCC), and non-small cell lung cancer (NSCLC).

### PD-L1 inhibitors

Three anti-PD-L1 antibodies have been approved by the FDA: atezolizumab (IgG4 mAb), durvalumab (IgG1 mAb), and avelumab (IgG1 mAb) [56]. Atezolizumab, as the first FDA-approved PD-LI inhibitor, was approved in 2016 to treat patients with advanced or metastatic urothelial carcinoma [57]. This anti-body also has been approved for patients with metastatic NSCLC whose disorder developed throughout or upon platinum-containing chemotherapy [58]. Moreover, the FDA approved atezolizumab plus bevacizumab, an antiangiogenic drug, for people with unresectable or metastatic HCC [59]. Finally, atezolizumab, in combination with mitogen-activated extracellular kinase (MEK) inhibitor cobimetinib and B-Raf enzyme inhibitor called vemurafenib, has been approved for patients with BRAF V600 mutation-positive unresectable or metastatic melanoma [60]. In 2017, durvalumab was first approved for the treatment of locally advanced or metastatic urothelial carcinoma [61] and also for metastatic Merkel cell carcinoma (MCC), a rare and aggressive skin cancer [62]. Durvalumab, in combination with etoposide and carboplatin or cisplatin, has been approved as the first-line treatment for patients with advanced NSCLC [63]. In 2017, avelumab was approved for MCC [62] and metastatic urothelial carcinoma therapy [64]. Moreover, FDA has approved avelumab in combination with tyrosine kinase inhibitor axitinib for the first-line treatment of patients with advanced RCC in 2019 [65].

## Monotherapy using immune checkpoint inhibitors in preclinical models

As described, six drugs targeting PD-1 or its ligand PD-L1 and one targeting CTLA-4 have been approved to treat diverse types of solid tumors and cHL. When used as monotherapy, the drugs mainly have a remarkable increase in objective response rate (ORR) and demonstrate a manageable safety profile. However, more than 50% of patients failed to respond to treatment. This section exclusively focuses on animal studies evaluating the therapeutic potential of ICIs therapy as monotherapy (Table 2).

**Table 2:** Monotherapy using immune checkpoint inhibitors (ICIs) in preclinical models (animal study)

Tumor	Target ICs	Main results	References
Glioma	CTLA-4	Induction of long-term survival in 80% of treated mice Reduction of CD4+ CD25+ Foxp3+ GITR+ Treg cell density	[69]
Mesothelioma	CTLA-4	Inhibition of tumor development at the early stage of tumor development Improving frequency of CD4 and CD8 T cells infiltrating the tumor	[73]
Hepatocellular carcinoma (HCC)	CTLA-4	Simulating longer survival in treated mice than control mice Amelioration of expression of CD4+ lymphocytes in residual tumors and IFN-γ generation	[74]
NA	CTLA-4	Inhibition of CD4+ CD25+ Treg function	[17]
Melanoma	CTLA-4	Augmentation of intratumoral T effector cell density in TME Reducing intratumoral Treg density in TME	[258]
Colon adenocarcinoma	CTLA-4	Enhancement of intratumoral T effector cell density in TME Plummeting intratumoral Treg density in TME	[70]
Colon adenocarcinoma	CTLA-4	Inspiring anti-tumor response by immune cell	[259]
Prostate cancer	CTLA-4	Modification of Treg activities is required for the anti-tumor impacts of the CTLA-4 blockade	[260]
Sarcomas	CTLA-4	Anti-tumor immunotherapy by CTLA-4 blockade depends on the gut microbiota	[261]
Melanoma	CTLA-4	Loss of IFN-γ axes in tumor cells is contributed to the cell resistance to anti-CTLA-4 therapy	[262]
Melanoma	CTLA-4	Suppression of melanoma stem cells tumourigenesis	[72]
Melanoma	PD-1/PD-L1	Tumors tempering the mitochondrial function in T cells show resistance to PD-1 blockade therapy	[263]
Oral squamous cell carcinoma (OSCC)	PD-1/PD-L1	Provoking the IFNγ, STAT1 activation and the making of the T-cell effector granzyme B in infiltrating cells Triggering apoptosis in the epithelial cells of the oral lesions	[82]
Pancreatic ductal adenocarcinoma (PDA)	PD-1/PD-L1	Mobilization of CD8+ T Cells by CXCR4 inhibition enables PD-1 checkpoint therapy	[86]
Myeloma	PD-1/PD-L1	Inhibition of tumor cell growth transiently	[84]
Melanoma	PD-1/PD-L1	Inhibition of tumor cell growth	[264]

Programmed cell death protein 1 (PD-1), programmed death-ligand 1 (PD-L1), cytotoxic-T-lymphocyte-associated protein 4 (CTLA-4), interferon-gamma (IFNγ), signal transducer and activator of transcription (STAT1), Forkhead box P3 (Foxp3), glucocorticoid-induced tumor necrosis factor receptor (GITR), regulatory T cells (Tregs), C-X-C chemokine receptor type 4 (CXCR4)

### CTLA-4 blockade

The constitutively expressed protein, CD28, arbitrates one of the best-recognized T cell costimulatory signals. CD28 binding to ligands B7-1 and B7-2 (CD80 and CD86) on APCs results eventually in T cell proliferation by stimulation of the production of interleukin-2 (IL-2) and anti-apoptotic factors [66]. CTLA-4 is expressed on activated T cells and has about 30% homologous with CD28 and also creates interaction with the ligands B7-1 and B7-2, thereby transmitting an inhibitory signal to T cells [67]. It has been found that monotherapy with antibodies to CTLA-4 can effectively instigate tumor regression of transplantable murine tumors. It seems that induction of effector T cells functions and suppressing Treg activities play central roles in this regard [17, 68].

Recently, studies in glioma xenograft models revealed that systemic administration of monoclonal antibody (9H10) to target CTLA-4 served prolonged survival in 80% of treated mice without exhibiting allergic encephalomyelitis [69]. Treatment caused diminished CD4+ CD25+ Foxp3+ GITR+ regulatory T cell fraction, supported improved CD4+ T-cell proliferative capacity, and provoked cervical lymph node antitumor response. These consequences indicated that CTLA-4 blockade could be rational modalities of restoring glioma-induced variations to the CD4 compartment and eliciting antitumor immunity [69]. Also, treatment with anti-CTLA-4 antibodies has shown promising anti-tumor effects in MC38 and CT26 tumor adenocarcinoma tumor models induced by selective attenuation of intra-tumor Treg with active T cell activation [70]. In addition, anti-CTLA-4 antibodies could ameliorate antitumor immunity through promoting melanoma-specific T-cell motility, according to Pentcheva et al. reports [71]. They suggested that CTLA-4 blockade could induce tumor immunity either by ameliorating effector T-cell activities or by depletion of Treg [71]. Importantly, treatment could give rise to improved T-cell motility, thereby supporting these T cells' enhanced frequencies in tumor-draining lymph nodes [71]. Remarkably, blocking CTLA‑4 in melanoma cells could also inhibit the particular competencies of melanoma stem‑like cells in vivo, comprising the capability for tumorigenesis [72]. CTLA-4 blocking antibodies also could hinder tumor growth at the early stage of murine mesothelioma [73]. Administration of anti-CTLA-4 antibody gave rise to an enhanced density of tumor-infiltrating CD4 and CD8 T cells. Also, it sustained the IL-2, IFN-γ, perforin, and granzyme B levels in TME in treated mice [73]. Moreover, anti-CTLA-4 therapy induced tumor regression and promoted survival after insufficient radiofrequency ablation (RFA) in the murine HCC model [74]. The analysis presented that expression of CD4+ T cells in residual tumors and IFN-γ generation in response to tumor cells were considerably higher in mice treated with anti-CTLA-4 than in the control group [74]. Other studies also have documented the importance of IFNγ in the CTLA-4 blockade-mediated anti-tumor response [75]. In a fibrosarcoma mice model, administration of anti-CTLA-4 anti-body caused raised levels of the IFN-inducible enzyme 2′,5′-oligoadenylate synthetase (OAS), a positive regulator of anti-tumor response, in draining lymph nodes concurrent with augmented levels of IFNγ in tumor lysates [75]. The prominence of IFNγ was confirmed through the aptitude of neutralizing antibodies to abolish the anti-tumor impacts of anti-CTLA-4 [75] wholly. In another study, Hanani et al. found that neutralizing IL-2 or blocking its receptor eliminated the antitumor effects and progression associated with the ratio of intra-tumor T effect versus Tregs, commonly induced by CTLA-4 blockade in melanoma mouse models [76]. In contrast, the administration of recombinant IL-2 led to the intensified therapeutic efficacy of CTLA-4 blockade [76]. The anti-CTLA-4 antibody also caused the abrogated Treg function and simultaneously improved IL-2-secreting effector T cell activities in vivo [76]. This study provides clear evidence illustrating the fundamental role of IL-2 and IL-2 receptors in the anti-tumor efficacy of CTLA-4 blockade [76]. Furthermore, Fransen et al. supposed that controlled local delivery of anti-CTLA-4 anti-body could trigger CD8+ T cell-dependent tumor elimination and reduced the risk of toxic side effects [77]. They found that lower dose and slow release of the anti-CTLA-4 anti-body gave rise to thousand-fold reduced levels of antibody in the serum, plummeting opposing events and the risk of autoimmunity in vivo [77]. Besides, they implied that CD4+ T cells do not play a noticeable role in the antibody-induced tumor regression [77].

### PD-1/PD-L1 blockade

Preclinical and clinical evidence have delivered the rationale for PD-1/PD-L1blockade as a millstone in cancer immunotherapy, rendering that induction of the PD-1/PD-L1 axis is respected as an efficient tool for tumor escape host tumor antigen-specific T-cell immunity. Indeed, the binding of PD-L1 on the tumor cells with PD-1 on a T-cell obstructs T-cell proliferation as well as activation and consequently restrain immune cell-mediated antitumor function [78].

Clear proof indicates that PD-1/PD-L1 blockade could ease T-cell migration to tumors by inspiring IFN-γ inducible chemokines like CTLA-4 inhibitors [79]. Meanwhile, Peng et al. found that anti-PD-1 antibody could not affect the frequency of immunosuppressive cells, such as Treg and myeloid-derived suppressor cells (MDSCs), during tumor progression in tumor-bearing mice. At the same time, it could augment the expression of IFN-γ and C-X-C motif chemokine ligand 10 (CXCL10) at the tumor zone [80]. Blocking the PD-1 pathway could stimulate IFN-γ at the tumor tissue, thus enhancing chemokine-dependent infiltration of immune cells into malignant disease zone [80]. Moreover, studies in BRAF^V600E^ mutation replace V600 valine-driven YUMM1.1 and YUMM2.1 melanomas, and the carcinogen-induced murine colon adenocarcinoma MC38 models clarified the critical competence of anti-PD-1 or anti-PD-L1 treatment to provoke potent antitumor effects versus tumor tissue in experimental models [81]. Anti-PD-1 therapy was also able to attenuate the number of oral lesions that developed in -nitroquinoline-1-oxide (4-NQO) mouse model of oral carcinogenesis and impede malignant progression in treated murine [82].

Meanwhile, low-grade dysplastic lesions reacted to anti-PD-1 therapy with a special promotion in the infiltration of CD8+ and CD4+ T cells and the accumulation of CTLA-4+ T cells in their TME [82]. In addition, PD-1 inhibition was associated with stimulation of IFNγ and signal transducer and activator of transcription 1 (STAT1) activation, production of granzyme B by infiltrating cells, and stimulation of apoptosis in the epithelial cells oral lesions [82]. These findings elucidated that T-cell activation arose from the anti-PD-1 therapy and suggested that CTLA-4 inhibitors may augment the preventive belongings of anti-PD-1 [82]. Monotherapy using anti-PD-1 or anti-PD-L1 antibody could also boost systemic T cell expansion, trigger objective responses, and convince the persistent neoantigen-specific T cell-mediated immunity in pancreatic ductal adenocarcinoma (PDA) murine model [83]. Furthermore, anti-PD-L1 administration suppressed myeloma cells' development in P815 tumor cell-bearing mice [84]. Also, transgenic expression of PD-L1 in P815 tumor cells maintained CTL-mediated tumor lysis in-vitro and increased tumorigenesis and invasiveness in-vivo [84].

Similarly, Zeng et al. disclosed that anti-PD-1 therapy-induced tumor regression inspired long-term survival of ovarian tumor-bearing mice [85]. They also found that AMD3100 could enhance these events, a particular C-X-C chemokine receptor type 4 (CXCR4), mainly by increasing the penetration and function of effective T cells, growing memory T cells in TME, and decreasing intratumoral Treg and MDSCs [85]. Thereby, it has been proven that PD-1 blocked therapy could be rational and practical candidates in ovarian cancer and could be clinically relevant to ovarian cancer patients [85]. Likewise, another report has shown that mobilization of CD8+ T cells through CXCR4 blockade supports anti-PD-1 therapy in PDA models in vivo [86].

## Predictive biomarkers in ICIs therapy

Though ICIs therapy has provided robust anti-tumor effectiveness, some patients do not show desired response to this therapeutic intervention [87]. Thus, more consideration has been compensated for identifying and advancing predictive biomarkers for ICI's reaction. Currently, with the progress of high-throughput sequencing and microarray methods, a diversity of biomarker plans have been discovered and offered the process from the detection of a single marker to the advance of multifactorial synergistic predictive markers [88]. Now, PD-L1 expression, high tumor mutational burden (TMB), microsatellite instability (MSI), CD8 infiltration, and PD-L1 amplification are considered as primary predictive markers for ICIs response [89–91].

TMB is the total number of mutations found in the DNA of cancer cells and is recently being used as a type of biomarker [92]. High numbers of mutations seem to be more probable to respond to certain types of immunotherapy [92]. Dramatic association between high TMB and response to ICIs have been verified in various cancer types, such as urothelial carcinoma [93], NSCLC [94, 95], melanoma [96, 97], human papillomavirus (HPV)-negative HNSCC [98], biliary tract cancer [99], small-cell lung cancer (SCLC) [100] and CRC cancers [101, 102]. For example, studies on 22 patients with metastatic CRC treated with PD-1/L1 inhibitors verified the existence of a close association between TMB and objective response. Meanwhile, all 13 TMB^high^ cases responded, while 6/9 TMB^low^ cases experienced progressive cancer [101]. On the other hand, any deregulation in mismatch repair (MMR) genes functions bring about high MSI (MSI-H), indicating a high amount of instability in the tumor. MSI-H tumors can mainly attract higher densities of TILs than low MSI (MSI-L), thereby showing a more favorable prognosis [103]. As cited, pembrolizumab has been approved in solid tumors with high MSI, based on a biomarker valuation of MSI status. High MSI correlates with the increased density of mutations in tumoral DNA, which is associated with more excellent rates of TMB and the enhanced presence of TILs and neoantigens [104]. Patients with MSI-H/deficient MMR tumors can usually benefit from ICIs therapy, and MSI can be applied as a genetic instability of a tumor detection index in various tumors, such as biliary tract cancer [99], NSCLC [90], gastroesophageal cancers [105, 106], breast cancer [104] and CRC [107]. Another study in 149 patients with endometrial cancer revealed that CD8+ T cells and PD-L1/PD-1 expression was considerably higher in the MSI group than in the microsatellite-stable group. Thereby, ICIs could be effective in endometrial cancers with MSI, and the existence of MSI may also be a biomarker for desired response to PD-1/PD-L1 immunotherapy [108]. Similarly, Xiao et al. suggested that MMR deficiency is accompanied by MSI phenotype, augmented TILs, and PD-L1 expression in immune cells in ovarian cancer [109]. Kumagai and coworkers have also supposed that PD-1 expression by CD8+ T cells and Treg cells negatively affect effector and immunosuppressive activities, respectively [110]. They pronounced that PD-1 blockade inspires both recovery of dysfunctional PD-1+ CD8+ T cells and improved PD-1+ Treg cell-elicited immunosuppression [110]. Given that a deep reactivation of effector PD-1+ CD8+ T cells rather than PD-1+ Treg cells by anti-PD-1 antibodies is required for tumor regression, they suggested that PD-1 expression could be used as a predictive biomarker for PD-1 blockade therapies [110]. Besides, the fact that ipilimumab plus nivolumab has been approved for the first-line treatment of patients with NSCLC whose tumors express PD-L1 (≥ 1%) [47], highlights the importance of the PD-L1 expression as a pivotal predictive biomarker in this regard. In addition, other studies have shown that PD-L1, LAG3, and indoleamine 2, 3-dioxygenase 1 (IDO1) expressions in TILs presented a more appropriate prognosis for patients with MSI-H colon cancer [111]. Also, MDM2/4 amplification (AMP) is correlated with hyperprogression during ICI therapy in various tumors types, in particular NSCLC, and predicts poor response to ICIs [112]. In sum, assessment of the combination of several parameters is of paramount importance for the successful prediction of the cancer patient’s responses to ICIs.

## Clinical trials

**Fig. 3:**
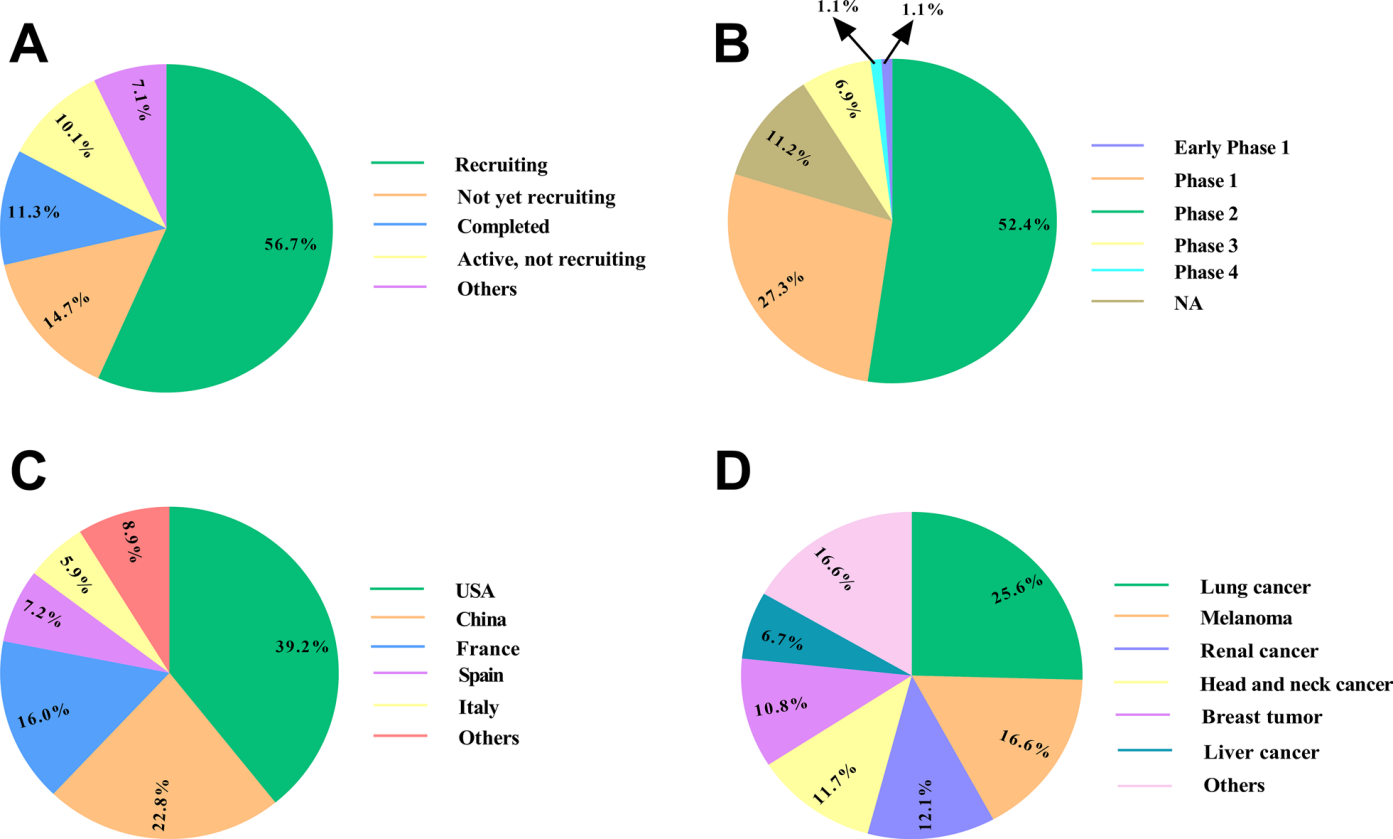
Clinical trials based on tumor immunotherapy using immune checkpoint inhibitors (ICIs) registered in ClinicalTrials.gov (November 2021). The schematic illustrates clinical trials using ICIs depending on the study phase (**A**), study status (**B**), conditions (**C**), and agents (**D**) in cancer patients

Approved ICIs are the anti-PD1 antibodies, including nivolumab, cemiplimab, and pembrolizumab; anti-PD-L1 antibodies comprised of atezolizumab, avelumab, and durvalumab; and the anti-CTLA-4 antibody called ipilimumab. Many trials have been accomplished or are ongoing to evaluate the safety and efficacy of ICIs in human tumors, including melanoma, NSCLC, RCC, MCC, CRC, cHL, urothelial cancer, and various deficient MMR/MSI-H solid tumors, etc. (Fig. 3) (Tables 3, 4).

**Table 3:** Clinical trials based on immune checkpoint inhibitors (ICIs) therapy in human malignancies registered in ClinicalTrials.gov (November 2021)

Condition	Drug	Phase	Participant number	Location	Status	NCT number
Hepatocellular carcinoma (HCC)	Camrelizumab Apatinib	2	40	China	Recruiting	NCT04826406
Oral squamous cell carcinoma (OSCC)	Avelumab	2	240	Italy	Recruiting	NCT04504552
Solid tumor Lymphoma	Nivolumab Pembrolizumab Atezolizumab Durvalumab	2	40	USA	Recruiting	NCT03544723
Brain cancer	Nivolumab	2	180	USA	Recruiting	NCT03173950
Squamous cell carcinoma of head and neck (SCCHN)	Nivolumab	2	24	USA	Recruiting	NCT03878979
Thymic carcinoma	KN046	2	29	USA	Not yet recruiting	NCT04925947
Gastric adenocarcinoma	Nivolumab	2	124	China	Recruiting	NCT04908566
Cholangiocarcinoma	Lenvatinib Sintilimab	2	25	China	Not yet recruiting	NCT05010681
Gastric cancer Liver cancer	IMC-001	2	48	S. Korea	Recruiting	NCT04196465
Non-small-cell lung carcinoma (NSCLC)	Durvalumab	2	55	USA	Recruiting	NCT04062708
Renal transitional cell carcinoma (TCC)	Pembrolizumab ramucirumab	2	28	USA	Recruiting	NCT04179110
HCC	Pembrolizumab Regorafenib	2	119	International	Recruiting	NCT04696055
Renal cell carcinoma (RCC)	Atezolizumab Cabozantinib	3	500	International	Recruiting	NCT04338269
Solid tumors	Nivolumab Pembrolizumab	1/2	104	USA Canada	Recruiting	NCT03311334
NSCLC Melanoma	Infliximab Vedolizumab	1/2	100	USA	Recruiting	NCT04407247
Pancreatic cancer	M7824	1/2	52	USA	Recruiting	NCT04327986
NSCLC	Pembrolizumab	1/2	30	China	Completed	NCT04670107
NSCLC	Atezolizumab Tocilizumab	1/2	28	USA	Not yet recruiting	NCT04691817
Advanced cancers	Nivolumab Ipilimumab Pembrolizumab	1/2	104	USA	Completed	NCT02467361
Prostate cancer	Pembrolizumab	2	100	UK	Recruiting	NCT03506997
NSCLC	Atezolizumab	2	21	USA	Active, not recruiting	NCT03689855
Gastric carcinoma	Toripalimab	2	70	China	Not yet recruiting	NCT04891016
RCC	Tivozanib Nivolumab	3	326	USA	Active, not recruiting	NCT04987203
NSCLC	Ipilimumab Nivolumab	3	1360	France	Recruiting	NCT03469960
Advanced cancers	INCB086550	2	150	Bulgaria Ukraine	Not yet recruiting	NCT04629339
Prostate cancer	Pembrolizumab	2	33	USA	Recruiting	NCT03406858
HCC	Cabozantinib	2	46	Italy	Recruiting	NCT04435977
Brain tumor	Pembrolizumab	2	30	USA	Recruiting	NCT04479241
SCCHN	Monalizumab Cetuximab	3	600	International	Recruiting	NCT04590963
Cervical cancer	Durvalumab	2	37	S. Korea	Not yet recruiting	NCT04800978
Melanoma	Nivolumab Ipilimumab	1/2	72	Australia	Recruiting	NCT03161756
Melanoma	Indoximod Ipilimumab Nivolumab Pembrolizumab	1/2	132	USA	Completed	NCT02073123
Solid tumor	Biological Nivolumab	1/2	102	USA	Recruiting	NCT04317105
Colorectal cancer	Atezolizumab	2	52	France	Recruiting	NCT04659382
Oesophageal cancer	Nivolumab Ipilimumab	2	130	France Spain	Recruiting	NCT03437200
HCC	Durvalumab	2	37	Hong Kong	Recruiting	NCT04913480
NSCLC	Camrelizumab	2	62	China	Recruiting	NCT04167774
Advanced cancers	Toripalimab	2	35	China	Recruiting	NCT03810339
Prostate cancer	Ipilimumab Nivolumab	2	75	USA	Recruiting	NCT04717154
NSCLC	Atezolizumab	4	100	S. Korea	Recruiting	NCT04059887
Pancreatic cancer	Pembrolizumab	2	16	Denmark	Recruiting	NCT04835402
Nasopharyngeal neoplasms	Camrelizumab	3	442	China	Recruiting	NCT03427827
Colorectal neoplasms Breast neoplasms	Durvalumab	2	384	USA	Recruiting	NCT02484404
Nasopharyngeal carcinoma	Toripalimab	3	494	China	Not yet recruiting	NCT04907370
Mesothelioma	Durvalumab	3	480	International	Recruiting	NCT04334759
Immune-mediated colitis	Tofacitinib	2	10	Canada	Not yet recruiting	NCT04768504
Anal cancer	Durvalumab	2	178	Germany Switzerland	Recruiting	NCT04230759
Metastatic solid tumor	Cemiplimab	2	38	Netherlands	Not yet recruiting	NCT04706715
Hepatocellular carcinoma Colorectal neoplasms Gastric cancer Lung cancer	Nivolumab pembrolizumab	2	80	USA	Recruiting	NCT03259867
Nasopharyngeal carcinoma	Durvalumab	2	118	Hong Kong	Recruiting	NCT04447612
NSCLC	Camrelizumab	2	40	China	Recruiting	NCT04541251
Esophageal cancer Metastatic cancer Squamous cell carcinoma	Cabozantinib atezolizumab	2	37	Taiwan	Recruiting	NCT05007613
Cervical cancer	Atezolizumab	2	189	France	Recruiting	NCT03612791
Pancreatic cancer	Pembrolizumab	2	24	USA	Completed	NCT03331562
Nasopharyngeal carcinoma	Toripalimab	2	126	China	Recruiting	NCT04517214
Breast cancer	Pembrolizumab	2	46	Germany	Recruiting	NCT03988036
Breast cancer	Pembrolizumab	2	15	Israel	Recruiting	NCT03591276
Solid tumors	Ipilimumab nivolumab pembrolizumab atezolizumab	2	60	USA	Recruiting	NCT03693014
Gastrointestinal cancer	Atezolizumab	2	175	USA	Recruiting	NCT04214418
Breast cancer	Nivolumab	2	90	S. Korea	Recruiting	NCT04061863
Solid tumors	Ipilimumab Nivolumab Pembrolizumab Atezolizumab Avelumab Durvalumab Cemiplimab	2	126	Netherlands	Recruiting	NCT04954599
NSCLC	Pembrolizumab	2	85	USA	Recruiting	NCT03233724

**Table 4:** The results of most important clinical trials based on immune checkpoint inhibitors (ICIs) therapy alone or in combination with other modalities in cancer patients

Condition	Agents	Result	References
Untreated melanoma	Ipilimumab + nivolumab	Nivolumab alone or combined with ipilimumab caused significantly longer PFS than ipilimumab alone	[125]
Advanced melanoma	Nivolumab + ipilimumab	This combination had a controllable safety profile and provided clinical activity	[265]
Advanced UC	Nivolumab + ipilimumab	This combination provided an effective treatment strategy	[266]
NSCLC	Nivolumab + ipilimumab + chemotherapy	This combination provided a significantly longer OS against chemotherapy alone	[213]
Resectable NSCLC	Atezolizumab + carboplatin + nab-paclitaxel	This combination achieving a major pathological response, and controllable treatment-related toxic effects	[267]
Urothelial cancer	Pembrolizumab	Pembrolizumab has become a new treatment choice	[130]
Colorectal cancer	Nivolumab	Nivolumab provided strong responses	[124]
NSCLC, melanoma, renal-cell cancer	Nivolumab	Nivolumab is caused in objective responses	[123]
Recurrent glioblastoma	Pembrolizumab	Pembrolizumab enhances both the local and systemic antitumor immune response	[129]
Incurable human papillomavirus 16-related cancer	Nivolumab + ISA101	This combination provided a clinical activity compared with nivolumab alone	[239]
Locally advanced and metastatic UC	Atezolizumab	Atezolizumab showed durable clinical activity and good tolerability	[268]
Unresectable hepatocellular carcinoma	Atezolizumab + bevacizumab	This combination made a longer PFS than with atezolizumab alone	[269]
NSCLC	Ipilimumab + radiation	This combination provided evidence that can be considered a treatment strategy	[270]
TNBC	Nivolumab + doxorubicin + cisplatin	They indicated that cisplatin and doxorubicin may increase the likelihood of response to nivolumab in TNBC	[271]
Extensive-stage small-cell lung cancer	Durvalumab + platinum-etoposide	This combination showed sustained OS improvement versus platinum-etoposide alone	[272]
NSCLC	Durvalumab + tremelimumab	This combination showed a controllable tolerability profile, with antitumor activity	[273]
Metastatic squamous cell carcinoma	Nivolumab	Nivolumab significantly improved OS	[119]
Resectable glioblastoma	Nivolumab	Nivolumab significantly improved OS	[120]
Advanced nonsquamous NSCLC	Nivolumab	Nivolumab significantly improved OS in patients that had progressed during or after chemotherapy	[121]
Advanced melanoma	Nivolumab and ipilimumab	Nivolumab plus ipilimumab or nivolumab alone significantly improved OS than ipilimumab alone	[274]
Recurrent squamous-cell carcinoma of the head and neck	Nivolumab	Nivolumab resulted in longer OS than treatment with standard, single-agent therapy	[122]
Advanced melanoma	Pembrolizumab against ipilimumab	The pembrolizumab prolonged PFS and OS and had less high-grade toxicity than did ipilimumab	[131]
Metastatic melanoma	Ipilimumab + glycoprotein 100 (Gp100)	This combination, as compared with gp100 alone, improved OS in patients	[275]
Squamous NSCLC	Pembrolizumab + chemotherapy	This combination resulted in significantly longer OS and PFS than chemotherapy alone	[276]
Metastatic NSCLC	Pembrolizumab + chemotherapy	This combination resulted in significantly longer OS and PFS than chemotherapy alone	[277]
Early TNBC	Pembrolizumab + chemotherapy	This combination resulted in a significantly higher pathological complete response than chemotherapy alone	[128]
Advanced UC	Pembrolizumab	This combination resulted in significantly longer OS than chemotherapy alone	[127]
Untreated metastatic nonsquamous NSCLC	Pembrolizumab + pemetrexed-platinum	This combination demonstrated substantially improved OS and PFS	[278]
MSI-H/dMMR noncolorectal cancer	Pembrolizumab	Pembrolizumab monotherapy demonstrated clinical benefits for the patients	[126]
Advanced CSCC	Cemiplimab	Cemiplimab induced a response in approximately half of the patients	[140]
Advanced CSCC	Cemiplimab	Cemiplimab showed antitumor activity and an acceptable safety profile	[139]
Metastatic CSCC	Cemiplimab	Cemiplimab produced substantial antitumor activity with a durable response and an acceptable safety profile	[138]
Advanced malignancies	Cemiplimab + radiotherapy and/or low-dose cyclophosphamide	Cemiplimab exhibited encouraging antitumor activity	[279]
Unresectable hepatocellular carcinoma	Atezolizumab + bevacizumab	Atezolizumab combined with bevacizumab resulted in better OS and PFS outcomes	[280]
NSCLC	Atezolizumab	Atezolizumab treatment resulted in significantly longer OS than platinum-based chemotherapy	[148]
NSCLC	Atezolizumab + bevacizumab + chemotherapy	This combination improved PFS and OS	[58]
Advanced TNBC	Atezolizumab + nab-paclitaxel	This combination prolonged PFS	[281]
Metastatic non-squamous NSCLC	Atezolizumab + carboplatin + nab-paclitaxel	This combination showed a significant and clinically meaningful improvement in OS and PFS	[282]
Early-stage TNBC	Atezolizumab + chemotherapy	This combination significantly resulted in pathological complete response rates with an acceptable safety profile	[283]
Metastatic urothelial cancer	Atezolizumab + chemotherapy	This combination prolonged PFS	[284]
Melanoma	Atezolizumab + vemurafenib, + cobimetinib	This combination significantly increased PFS and it was tolerable and safe	[285]
Advanced or metastatic UC	Avelumab	Avelumab with best supportive care significantly prolonged OS, as compared with best supportive care alone	[64]
Metastatic UC	Avelumab	Avelumab showed antitumor activity in the treatment of patients	[156]
Advanced or metastatic breast cancer	Avelumab	Avelumab exhibited a clinical activity and acceptable safety profile	[153]
Recurrent or refractory ovarian cancer	Avelumab	Avelumab demonstrated antitumor activity and acceptable safety	[155]
Relapsed or refractory extranodal NK/T-cell lymphoma	Avelumab	Avelumab showed single-agent activity	[154]
Advanced GC/GEJC	Avelumab + chemotherapy	Avelumab showed a more controllable safety profile than chemotherapy alone	[286]
NSCLC	Durvalumab	Durvalumab prolonged PFS than with placebo	[158]
NSCLC	Durvalumab	Durvalumab monotherapy caused significantly longer OS than placebo	[159]
NSCLC	Durvalumab	Durvalumab demonstrated durable PFS and sustained OS after chemoradiotherapy	[160]
Extensive-stage small-cell lung cancer (ES-SCLC)	Durvalumab + tremelimumab + platinum	Durvalumab plus platinum-etoposide demonstrated sustained OS improvement against platinum-etoposide alone	[287]
Recurrent or metastatic cervical cancer	Cemiplimab + radiation therapy	Cemiplimab demonstrated clinical activity	[288]
Advanced melanoma, NSCLC, bladder cancer	Nivolumab + NEO-PV-01	This combination therapy was safe and feasible	[289]
Melanoma	Pembrolizumab + oncolytic virotherapy	The addition of oncolytic virotherapy might improve the value of pembrolizumab by changing the tumor microenvironment	[290]
Melanoma	Ipilimumab + talimogene laherparepvec	This combination was tolerated safely	[291]

Non-small cell lung cancer (NSCLC), urothelial cancer (UC), progression-free survival (PFS), overall survival (OS), mismatch repair (MMR); high microsatellite instability (MSI-H), triple-negative breast cancer (TNBC), gastric cancer/gastro-oesophageal junction cancer (GC/GEJC), cutaneous squamous-cell carcinoma (CSCC)

### CTLA-4 inhibitors

Ipilimumab is the most prominent member of the CTLA-4 inhibitors and also is defined as the first FDA-approved ICI. It has been approved for melanoma, MSI-H/dMMR CRC, intermediate or poor-risk RCC (in combination with nivolumab).

Based on trials recorded at ClinicalTrials.gov, 610 studies have been evaluating the antitumor effects of ipilimumab in participants suffering from melanoma, RCC, CRC, myeloma, NSCLC, glioblastoma, liver cancer, and prostate cancer. Of those, 62 ongoing studies are in phases 3 or 4. Among 610 studies, five trials (melanoma, lung cancer, kidney cancer, and RCC) are in phase 4. In addition, to strengthen the efficacy of ipilimumab in combination with nivolumab in NSCLC, this regimen could be an effective option for improving the median OS in patients with malignant pleural mesothelioma (MPM) [48]. Meanwhile, a phase 3 study (CheckMate 743) on 750 MPM patients reported the superiority of combination therapy with nivolumab (systemic 3 mg/kg) plus ipilimumab (1 mg/kg) on platinum plus pemetrexed chemotherapy in terms of improved median OS [48]. The median OS was 18.1 months in patients receiving combination treatment versus 14.1 months inpatient treated with a chemotherapy agent. Also, 2-year OS rates were 41% versus 27% in the nivolumab plus ipilimumab group and chemotherapy group, respectively. The results of this study supported the application of this regimen for previously untreated unresectable MPM from October 2020 [48].

Further, combination therapy with ipilimumab and nivolumab exhibited a more favored effect on PFS rate in patients with stage III or stage IV melanoma than monotherapy A 4 years follow-up showed a median PFS of 11.5 months in the nivolumab plus ipilimumab group, 6.9 months in the nivolumab group, and 2.9 months in ipilimumab group [113]. Further, the most common treatment-associated grade 3 side effects were diarrhea in the co-treated group (9%) and the nivolumab group (3%) and also colitis in the ipilimumab group (7%). Additionally, enhanced lipase (3–5%) was the most common grade 4 side effect in all three groups. The achieved outcomes demonstrated a durable and sustained survival benefit in patients with advanced melanoma who received ICIs alone or in combination [113].

Tremelimumab is another well-known CTLA-4 inhibitor. Unlike ipilimumab which is an IgG1 isotype, tremelimumab is an IgG2 isotype. According to trials registered in ClinicalTrials.gov, there exist 164 studies based on tremelimumab therapy in participants with various tumors. Meanwhile, 12 of them, including NSCLC, bladder cancer, head, and neck squamous cell carcinoma (HNSC), urothelial cancer, and HCC are in phase 3; however, there is no registered study in phase 4. In addition, a phase 2 study of tremelimumab (15 mg/kg intravenously) in advanced uveal melanoma patients who had not received prior immunotherapy demonstrated the safety and acceptable efficacy with survival benefits (median OS about 12.8 months) [114]. Also, administration of the tremelimumab (1 mg/kg) in combination with anti-PD-L1 monoclonal antibody durvalumab (20 mg/kg) elicited promising outcomes in mesothelioma patients [115]. The intervention caused improvement in median PFS (5.7 months) and median OS (16.6 months) irrespective of PD-L1 expression levels [115]. However, 18% of patients experienced high grade 3–4 treatment-associated adverse events. Thereby, it seems that comprehensive dose-escalation studies are required prior accomplishment of large scale phase 2 and 3 trials [115]. Furthermore, Pakkala et al. (2020) exhibited that combination therapy with durvalumab and tremelimumab could not be an effective therapeutic regimen for relapsed SCLC, highlighting the importance of the conduction of further studies to justify designing and conduction of phase 3 trials [116]. Besides, quavonlimab (MK-1308), a novel anti-CTLA-4 antibody, in conjunction with pembrolizumab, resulted in ORR about 40.0% in advanced NSCLC patients [117]. Importantly, PD-L1 expression and a total number of circulating CD4+ cells associated with ORR [117].

### PD-1 inhibitors

As a human IgG4 monoclonal antibody, Nivolumab is the second FDA-approved systemic treatment for mesothelioma and the first-line FDA-approved immunotherapy for gastric cancer [118]. Regarding trials registered in ClinicalTrials.gov, there are 1152 documented studies conducted or ongoing to address nivolumab's safety and efficacy in many tumors, 115 of which (melanoma, NSCLC, mesothelioma, breast cancer, RCC, lymphoma, HCC, urothelial cancer, etc.) are in phases 3 or 4 while 6 of which (NSCLC, kidney cancer, and RCC) are in phase 4. There have been several studies in the literature reporting that the nivolumab monotherapy has beneficial effects in the treatment of patients with metastatic squamous cell carcinoma, glioblastoma, NSCLC, melanoma, colorectal cancer, renal cell cancer, and recurrent squamous-cell carcinoma of the head and neck [119–124] (NCT02105636) (NCT02550249) (NCT01673867) (NCT02105636) (NCT00730639) (NCT02060188). The findings of these studies confirm the association between PD-L1 gene expression in tumor cells and the objective responses. Larkin et al. showed that using nivolumab alone or combined with the ipilimumab resulted in a significantly longer PFS than ipilimumab alone in patients with unresectable stage III or IV melanoma [125] (NCT01844505). The results of this study showed that the combination of nivolumab and ipilimumab had more beneficial effects in patients with PD-L1 negative tumors than in patients treated with either of these drugs alone [125].

Pembrolizumab, another PD-1 inhibitor, has been approved for cervical cancer, gastric cancer, HNSCC, HCC, cHL, melanoma, MCC, NSCLC, diffuse large B-cell lymphoma (DLBCL), and urothelial cancer. Regarding trials registered in ClinicalTrials.gov, 1355 registered trials assess the safety and efficacy of pembrolizumab in human cancers. The 140 (NSCLC, RCC, lymphoma, HCC, endometrial cancers, melanoma, biliary tract carcinoma, urothelial cancer, etc.) are in phases 3 or 4. 4 trials (NSCLC, melanoma, thymoma and thymic carcinoma, and SCC) are in phase 4. Pembrolizumab monotherapy has shown a survival benefit for several cancers, including Recurrent glioblastoma, early TNBC, advanced urothelial cancer (UC), and MSI-H/dMMR solid tumors (NCT02628067) (NCT03036488) (NCT02256436) [126–129]. Balar et al. reported that pembrolizumab had become a new treatment strategy for UC patients [130] (NCT02335424). The other study done by Robert et al. demonstrated that the pembrolizumab prolonged PFS and OS and had less toxicity than ipilimumab in patients with melanoma [131] (NCT01866319). During Phase 2, 259 patients with advanced gastrointestinal or esophageal cancer received 200 mg of pembrolizumab intravenously. Pembrolizumab monotherapy exhibited auspicious activity (durable responses) and a manageable safety profile in these patients [132]. Pembrolizumab also was well tolerated and elicited significant antitumor effects in patients with BCG-unresponsive non-muscle-invasive bladder cancer [133]. The standard grade 3 or 4 treatment-related adverse events were arthralgia (2%) and hyponatremia (3%) [133]. Thereby, pembrolizumab monotherapy can be suggested as a potential non-surgical therapeutic approach in difficult-to-treat bladder cancer patients. In addition, pembrolizumab monotherapy was associated with durable antitumor effects in metastatic triple-negative breast cancer (mTNBC) patients [134]. The ORR and disease control rate (DCR) was significantly but not strongly higher in the PD-L1-positive populations than the total population [134]. These results heightened the importance of defining and assessing predictive biomarkers before conducting ICIs therapies to distinguish between responder and non-responder patients [134]. Significantly, systemic administration of pembrolizumab 200 mg every three weeks improved PFS in HL patients who have relapsed post-autologous HSCT [135]. In high-risk stage III melanoma, pembrolizumab promoted 3.5-year distant metastasis-free survival and recurrence-free survival more efficiently in PD-L1-positive tumors [136]. Hence, the outcome of this phase 3 trial supported the indication to exploit adjuvant pembrolizumab therapy in advanced cutaneous melanoma patients. Similar results were obtained in patients with kidney cancer at high risk for recurrence following pembrolizumab monotherapy [137].

Another PD-1 inhibitor, cemiplimab, has been approved for cutaneous SCC. 1355 registered trials assess the safety and efficacy of cemiplimab in various human tumors. There are 55 documented studies based on cemiplimab therapy for human tumors, of which 3 of them (cutaneous SCC and NSCLC) are in phase 3, while there is no registered trial in phase 4. The cemiplimab monotherapy also provided a survival benefit and acceptable safety in patients with advanced and metastatic cutaneous squamous cell carcinoma (CSCC) [138–140] (NCT02383212 and NCT02760498) (NCT02760498) (NCT02760498). Among patients with advanced CSCC, cemiplimab stimulated a response in approximately half the patients and was associated with severe events that usually occur ICIs [138–140]. Cemiplimab monotherapy (3 mg/kg intravenously) encouraged antitumor effects due to its 44% objective response (34 out of 78 patients) and an acceptable safety profile in patients with CSCC [139]. Indeed, 10 patients exhibited complete response, and 24 participants displayed partial response, suggesting cemiplimab as a potential treatment option for CSCC therapy [139]. The promising results of this study and similar studies led to the approval of this cemiplimab for CSCC therapy in September 2018. Another phase 3 trial also signified the more prominent anti-tumor effects of cemiplimab monotherapy versus chemotherapy in NSCLC patients with PD-L1 of at least 50% [141]. Accordingly, the median PFS was 8.2 months versus 5.7 months in the cemiplimab group versus with chemotherapy group [141]. Further, serious adverse events happened in 28% of patients who received cemiplimab ad 39% of patients in the chemotherapy group. Thereby, in terms of safety and efficacy, it appears that cemiplimab is a more favored treatment than conventional chemotherapies. Thus, it seems that cemiplimab can receive approval from FDA for NSCLC with PD-L1 of at least 50% [141].

Sintilimab, a humanized IgG4 anti-PD-1 monoclonal antibody, is one of the most important PD-1 inhibitor that has not yet been approved by FDA despite promising clinical results. Sintilimab administration (200 mg/patient intravenously) has exhibited major pathologic response (MPR) (40.0%) and also objective response (20.0%) in NSCLC patients [142]. Also, in another phase 1b trial (NCT03628521) on 22 NSCLC patients, it improved median PFS rate to 15 months with no grade 4 treatment-related adverse events [143]. In addition, tislelizumab (BGB-A317), a humanized IgG4 anti-PD-1 monoclonal antibody, has shown anti-tumor potential in advanced solid tumors more evidently at the 5 mg/kg dose [144]. With respect to the observed results, various studies are underway to prove the safety and efficacy of tislelizumab in patients suffering from ESCC, gastric cancer, HCC, lung cancer and UC [144].

Recently, MDX-1106, a humanized IgG4 anti-PD-1 monoclonal antibody, as another type of the PD-1 inhibitors exhibited antitumor activity in metastatic melanoma, CRC, castrate-resistant prostate cancer (CRPC), NSCLC and RCC patients [145]. As well, there is clear evidence indicating the beneficial effects of administration of prolgolimab [146] and toripalimab [147], two other types of the anti-PD-1 monoclonal antibody, in melanoma patients [146] and also in patients with chemorefractory metastatic nasopharyngeal carcinoma (NPC) [147], respectively. Nonetheless, the preliminary results have to be validated in the large scale phase 2 and 3 trials.

### PD-L1 inhibitors

Atezolizumab is a human IgG1 approved for NSCLC and urothelial cancer. There are 488 trials based on atezolizumab therapy in the context of tumor therapy. 84 trials are in phases 3 or 4, and only 4 (NSCLC) are in phase 4. In a study conducted by Herbst et al., the efficacy and safety of the atezolizumab were investigated compared with the use of platinum-based chemotherapy [148]. Examination of the safety of atezolizumab in 615 patients with advanced NSCLC verified the benefit-risk profile of atezolizumab monotherapy in these patients [149]. Atezolizumab monotherapy also was well-tolerated in UC patients and resulted in an objective response of about 40% of patients with PD-L1 expression of at least 5% tumor-infiltrating immune cells [150]. Thereby, atezolizumab may offer durable anti-tumor activity in UC patients; however, further studies are warranted. Likewise, systemic administration of atezolizumab 1–20 mg/kg or 1200 mg led to objective response up to 50% of NSCLC patients with acceptable safety profile [151]. Notably, the ameliorated responses and survival rates were realized with augmenting baseline PD-L1 expression as expected [151].

Another PD-L1 inhibitor, avelumab, has been approved for MCC and UC. In 2019, the FDA approved avelumab combined with axitinib for the first-line treatment of people with advanced RCC [152]. Among 184 registered trials, 17 studies are in phase 3 (NSCLC, MCC, ovarian cancer, SCC, DLBCL, and RCC), while no study is in phase 4. Powles et al. used the avelumab in patients with UC and showed that the avelumab had a tolerable safety profile and clinical activity among these patients [64] (NCT02603432). Also, avelumab significantly improved the OS rate among patients with advanced or metastatic breast cancer [153] (NCT01772004). Moreover, Kim et al. determined the effect of avelumab in patients with relapsed or refractory extranodal natural killer/T-cell lymphoma [154] (NCT03439501). They found that the response to the avelumab was affected by the expression of PD-L1 in tumor tissues. They emphasized that assessing PD-L1 expression in tumor cells can help identify the responders to the PD-L1 antibody [154]. Also, this antibody has shown a significant antitumor effect and acceptable safety in patients with recurrent or refractory ovarian cancer [155, 156].

Durvalumab is another FDA-approved PD-L1 inhibitor, a monoclonal antibody of isotype IgG1, verified for NSCLC and urothelial carcinoma [61, 157]. Among 508 registered trials for this ICI, 47 studies are in phases 3 or 4, and 5 studies (NSCLC) are in phase 4. Antonia et al. assessed the durvalumab as consolidation therapy after the chemoradiotherapy in stage III NSCLS chemoradiotherapy (NCT02125461) (NCT02125461). This antibody made a significantly longer PFS in patients receiving the durvalumab than in patients receiving placebo [158, 159]. Also, durvalumab as consolidation therapy after the chemoradiotherapy has supported promising outcomes in patients with stage III NSCLC [160] (NCT02125461). Meanwhile, durvalumab monotherapy (10–20 mg/kg) was well-tolerated in Japanese patients with advanced solid tumors [161]. However, a significant anti-tumor effect was not observed [161]. Nonetheless, its administration into 70 patients with endometrial cancer provided an objective response in 47% of treated patients [162]. Also, the regimen led to improvement in OS rate12-month approximately 71% in endometrial cancer patients with dMMR with some managable grades 1–2 adverse events [162]. These findings delivered proof of the concepts that targeting PD-1/PDL-1 interaction can be a rational and effective therapeutic strategy for endometrial cancer. Notably, the higher antitumor efficacy of durvalumab administration in cancer with dMMR compared with cancers with MMR-proficient (MMRp) elucidates the robust rationale behind the application of immunotherapy in these subgroups of patients [162]. PD-L1 expression levels also impact therapeutic outcomes following durvalumab therapy, apart from MMR status. In this light, a recent phase 2 trial (NCT02207530) in recurrent/metastatic (R/M) HNSCC patients indicated that patients with ≥ 25% of tumor cells expressing PD-L1 exhibited favored responses to durvalumab therapy (10 mg/kg intravenously) [163]. Significant antitumour activity (e.g., ORR about 16.2%) along with acceptable safety profile in PD-L1-high patients with R/M HNSCC justified durvalumab ongoing evaluation in phase III trials [163]. Because the HPV-positive patients displayed better response and survival to durvalumab therapy than HPV-negative patients, it is highly recommended that the types of cancer (HPV-related HNSCCs and HPV-unrelated HNSCCs) be assessed before selecting patients for treatment irrespective of the PD-L1 expression patterns and MMR status [163].

Other PD-L1 inhibitors like envafolimab are in phase 1 and 2 clinical trials. Envafolimab is a first-in-class nanobody which its efficacy and also safety has recently been evidenced following subcutaneous injection in previously treated advanced dMMR/MSI-H solid tumors [164]. It was suggested that envafolimab could be an alternative to systemic administration of PD-1/PD-L1 inhibitors for advanced, refractory solid tumors therapy [165]. BMS-936559 (NCT01455103) and SHR-1316 (HTI-1088) (e.g., NCT04647357, NCT03474289, NCT05082545) (two fully humanized IgG4 monoclonal antibodies), and also CK-301 (NCT03212404 and NCT04786964), BGB-A333 (NCT03379259), CBT-502 (TQB-2450) (e.g., NCT05111366, NCT05013697, NCT04665609) and CS-1001 (e.g., NCT03744403, NCT04472858, NCT03789604), which are fully human monoclonal antibody of IgG1, are other PD-L1 inhibitors, which evaluation of their safety and efficacy is being studied. Meanwhile, SHR-1316 administration demonstrated promising outcomes in esophageal squamous cell carcinoma (ESCC) [166]. Of course, most ongoing studies are related to CBT-502, while the results are not yet available.

## Current challenges

Although ICIs are at the forefront of immunotherapy for various cancers, they fail to modify tumor progress in a significant proportion of patients or arouse several serious adverse events.

### Toxicities of immune checkpoint inhibitors

Unfortunately, ICIs therapy has also been associated with the occurrence of some immune-related untoward events, which diverge among patients based on the agent, malignancy, and individual susceptibilities [167]. Skin and colon are the most mutual organs, while the liver, lungs, kidneys, and heart are negatively affected by ICIs. Invariably, such toxicities are detected by excluding other secondary infectious or inflammatory underlies [168]. Corticosteroids are generally utilized to alleviate moderate and severe immune-related unwanted events, whereas additional immunosuppressive modalities may sometimes be required [169, 170]. The incidence of such toxicities may necessitate cessation of immunotherapy regarding the specific toxicity and its severity.

CTLA-4 adjusts the competence of immunologic response at early stages of T-cell induction, while PD-1 and PD-L1 pathways perform at later stages, preventive T-cell function in the peripheral tissues [171, 172]. Such differences partially explain anti-CTLA-4, anti-PD-1, and anti-PD-L1 [172, 173]. Overall, common ICIs related toxicities includes systemic toxicities (fatigue, fever, chills, and infusion reactions), dermatological toxicities (rash or pruritus), gastrointestinal (GI) toxicities (diarrhea, hepatitis and colitis), endocrine toxicities (thyroid dysfunction, hypophysitis, adrenal insufficiency and type 1 diabetes mellitus), pulmonary toxicities (dyspnea, cough, wheezing, and increased supplemental oxygen requirement), rheumatologic toxicities (inflammatory arthritis, inflammatory myositis, rhabdomyolysis, giant cell arteritis, and polymyalgia-like syndrome), neurologic toxicities (motor or sensory peripheral neuropathies, myasthenia gravis-like syndrome, aseptic meningitis, autoimmune encephalitis, posterior reversible encephalopathy syndrome, and transverse myelitis), ocular toxicities (conjunctivitis, episcleritis, keratitis, blepharitis, and uveitis), renal toxicities (acute interstitial nephritis, lupus-like nephritis, granulomatous nephritis, diffuse interstitial nephritis), cardiac toxicities (myocarditis, pericarditis, arrhythmias and heart block) and also hematologic toxicities (anemia) [29, 173].

Based on recent reports, the occurrence of extreme immune-related adverse events (irAEs) has been observed to be as high as 27% with the use of CTLA-4 blockade in comparison to 16% with PD-1 blockade. It may improve to 55% once both therapies are applied concomitantly [125]. The incidence of specific toxicities diverges according to the types of malignancy or used ICI. Meanwhile, patients with melanoma seem to demonstrate higher degrees of rash and colitis and lower pneumonitis than RCC and NSCLC [174]. Moreover, CTLA-4 blockade may result in higher degrees of colitis, hypophysitis, and inflammation, while pneumonitis, thyroiditis/hypothyroidism, arthralgias, and vitiligo are associated with PD-1 blockade [174, 175]. Notably, irAEs are dose associated with anti-CTLA-4 antibodies but not anti-PD-1 antibodies [176].

### Tumor resistance to ICIs

Nowadays, it is universally accepted that transformed cells shape tight interactions with the extracellular matrix (ECM), stromal cells, and immune cells composing TME. These components of the TME organization facilitate a chronic inflammatory, immunosuppressive, and pro-angiogenic intratumoral milieu, which ultimately supports cancer cell escape and eradication by the host immune system [177, 178]. DCs must properly activate T cells in the peripheral lymph nodes to eliminate cancer cells in the tumor site and penetrate barriers (such as stromal tissue) [179, 180]. Emerging cancer frequently avert such requirements for T cell immunosurveillance to deter immune-mediated tumor regression. Because ICIs therapy's efficacy is primarily motivated by T cells, this efficient immune escape can finally underlie failures in ICIs treatment. A spectrum of studies has implied that upregulation of PD-L1 in the TME by cancer cells and APCs is one the most shared strategy by which cancers circumvents immune surveillance [181, 182]. Besides, the catabolism of tryptophan inside the TME plays an influential role in suppressing anti-tumor immune reactions. The IDO often catabolizes tryptophan in myeloid cells and tumor cells to produce immunosuppressive metabolites such as kynurenine [183]. The kynurenine activities associated with the exhaustion of the crucial amino acid tryptophan are broadly complicated in obstruction of clonal expansion of T cells. Also, they can provoke T cell anergy and apoptosis [183]. Therefore, a combination of IDO inhibitors and treatment of ICIs has been proposed to enforce TILs and their functional capabilities in TME and thus eradicate either IDO-expressing or nonexpressing poorly immunogenic cancer cells [184]. Some clinical trials are being conducted to address the safety and efficacy of IDO blockade plus ICIs (NCT02073123, NCT01604889, and NCT02327078). Besides, the presence of Treg cells, Th2 cells, and MDSCs is another pivotal obstacle to ICIs therapies by inhibition of ICI-mediated anti-tumor CTL and Th1 cell responses [185, 186]. Exhaustion of such cell types, as might be plausible for Treg cells [187], can be employed together ICIs therapies to offer more appropriate outcomes. In addition, activation of some oncogenic factors, such as the WNT-β-catenin signaling pathway, severely inhibits the infiltration of TILs and CD103+ DC into TME by suppressing β-catenin-associated CCL4 chemokine in melanoma, thereby facilitating tumor escape [188, 189]. These reports have outlined the importance of developing an innovative plan for combination therapy with ICIs and other therapeutic modalities.

Expression of cyclooxygenase (COX) at high levels is another mechanism contributed to immune suppression in TME by the production of prostaglandin E2 (PGE2), which offers an inflammatory environment favoring tumor proliferation [190, 191]. Assessment of tumor and immune cell glucose and glutamine metabolism has led to the raising evidence signifying that the metabolic interaction between the tumor cells and immune cells can sustain the poor response to ICIs [192]. Indeed, such metabolism promotes the expression of PD-L1 in tumor cells by the epidermal growth factor receptor (EGFR)/extracellular signal-regulated kinase (ERK)/C-Jun pathway [192]. Thereby, the targeting of tumor glucose or glutamine metabolism in combination with PD-1/PD-L1 targeting looks to serve new therapeutic chances for patients with tumors.

## Combination therapy with ICIs

The success of ICIs in patients with a diversity of human malignancies has evolved tumor immune therapies. At the same time, combination therapies with ICIs are always required to circumvent resistance and expand the clinical application of immunotherapy. Making crucial progress in combination therapy with ICI is of paramount importance given that only a small proportion of patients respond to ICI therapy, and many will relapse [193, 194]. Numerous clinical trials have been conducted to address the safety and efficacy of ICI in combination with conventional cancer therapies, targeted molecular compounds, and new immunomodulatory therapies [195]. In addition to the combination therapy with other therapeutic modalities, the combination of CTLA-4 and PD-1 blockers has been suggested to have a synergistic impact on eliciting the anti-tumor activities and consequently lessening the response rates in cancer patients [196, 197]. The therapeutic efficacy of combinational schemes has been determined by recent FDA approval of nivolumab plus ipilimumab for patients with advanced melanoma [198]. In this regard, the nominated combination therapy regimens include nivolumab plus ipilimumab (e.g., NCT02905266, NCT02998528, NCT02872116, and NCT02477826), pembrolizumab plus ipilimumab (e.g., NCT03302234, NCT04571632, and NCT04571632), atezolizumab plus ipilimumab (NCT02174172 and NCT04084951), and tremelimumab plus durvalumab (NCT02701400 and NCT02516241). Such trials mainly have concentrated on survival and other treatment response indices. At the same time, a restricted number of them explore the safety profile and maximum tolerable dose (MTD) of combination therapy protocols [198, 199]. Also, various studies have inspected combination ICI with traditional treatment means and plans such as chemotherapy, radian therapy, angiogenesis-inhibitors, and cancer vaccines (e.g., oncolytic viruses).

### Chemotherapy

Several investigations have been conducted or executed to evaluate the therapeutic potential of combination therapy with chemotherapy and ICIs. Also, a large number of studies have evidenced the therapeutic capacity of ICI plus chemotherapeutic agents, including cyclophosphamide [200, 201], fluorouracil [200], gemcitabine [202, 203], doxorubicin [204], oxaliplatin [205, 206], cis-platin [204, 207], paclitaxel [208], methotrexate [204] and vinblastine [204] in preclinical models (Table 5). It seems that chemotherapy with ICI may result in improved activation of APC, which elicits T-cell-related antitumor effect leading to the suppressed metastatic tumor growth, reducing the immunosuppressive M2 macrophage, Tregs and MDSCs population, and finally improving the expression of the IFNβ, and CCL5 and CXCL10 [204, 207, 209, 210]. Such events promote the OS rate in treated animals by enhancing the host immune system's recognition and eradication of tumor cells and concurrently condenses the immunosuppressive TME. Based on the result achieved from clinical trials, combination therapy is also mainly well-tolerated, and several trials have attained durable responses [211]. Though chemotherapy and ICB are mainly used concomitantly and at full doses, some trials have assessed the optimal dose or sequence of administration [211]. In 2018, the FDA approved atezolizumab combined with bevacizumab, paclitaxel, and carboplatin for the first-line treatment of patients with NSCLC [212]. Approval was concerning the open-label phase 3 study (NCT02366143) on 1202 patients with NSCLC who had not previously received chemotherapy [58]. As cited in previous sections, Socinski et al. showed that atezolizumab in combination with bevacizumab, paclitaxel, and carboplatin considerably promoted PFS and OS among patients with metastatic NSCLC [58]. Another randomized, open-label, phase 3 trial (NCT03215706) has also shown that nivolumab plus ipilimumab with two cycles of chemotherapy may offer an acceptable improvement in OS compared with chemotherapy alone and also has a suitable risk–benefit profile in NSCLC patients [213]. As well, some other trials based on ICI plus chemotherapeutic agents, such as cisplatin and pemetrexed or docetaxel, are being carried out on patients with NSCLC, gastric carcinoma, head and neck cancer, and also human papillomavirus-associated oropharyngeal squamous cell carcinoma (HPV(+)OPSCC) (e.g., NCT04945200, NCT04997382, NCT04062708, NCT04867330, NCT03532737, NCT04908566, and NCT04891016).

**Table 5:** Immune checkpoint inhibitors (ICIs) combination therapy with chemotherapy (animal study)

Tumor	ICI type	Chemotherapeutic agent	Main result	References
Breast tumor Ovarian tumor	PD-L1	Cyclophosphamide	Selective depletion of Treg in the tumor tissue in vivo	[201]
Breast tumor Lymphoma	PD-L1	Cyclophosphamide Fluorouracil Vinorelbine	Activation of circulating and tumor-infiltrating immune cells in vivo	[200]
Breast cancer	PD-1	Cyclophosphamide Vinorelbine	Activation of APC, and eliciting T-cell-related effect leading to the suppressed metastatic tumor growth in vivo	[209]
Pancreatic ductal adenocarcinoma (PDA)	PD-1	Gemcitabine	Restoring the tumor cell sensitivity to ICI in vivo	[202]
Mesothelioma	PD-1	Gemcitabine	Hindrance of tumor development in vivo Improving the overall survival of treated models in vivo	[203]
Lewis lung carcinoma (LLC)	PD-1	Gemcitabine	Arousing strong anti-tumor effect in vivo	[292]
Colon cancer Bladder cancer	PD-1 PD-L1	Methotrexate Vinblastine Doxorubicin Cis-platin Cyclophosphamide	Convincing robust anti-tumor response in vivo	[204]
Colon cancer Renal carcinoma	CTLA-4	Cyclophosphamide	Augmentation of the antitumor effect of anti-CTLA-4 therapy in vivo	[293]
Pancreatic cancer	PD-1	Gemcitabine	Enhancing the anticancer effect of M1 macrophages and the Th1 response in vivo	[294]
Small cell lung cancer (SCLC)	PD-L1	Gemcitabine	Restoring the antitumorigenic CD8+ cytotoxic T cells, dendritic cells, and M1 macrophage populations in vivo Reducing the immunosuppressive M2 macrophage and MDSCs population in vivo Increasing the expression of the IFNβ, and CCL5 and CXCL10 in vivo	[210]
Lung cancer	PD-L1	Oxaliplatin	Activation of dendritic cells (DCs CD80+ CD86+) and CD8+ T cells resulted in tumor regression in vivo	[206]
Colon cancer	PD-1	Cis-platin Oxaliplatin	Triggering T cell activation and recruitment into tumors in vivo	[205]
Triple-negative breast cancer (TNBC)	PD-L1	Paclitaxel	Provoking the tumor regression, and inhibition of tumor metastasis in vivo	[208]
TNBC	PD-L1	Paclitaxel	Inducing the TILs infiltration into TME in vivo	[295]
Fibrosarcoma	PD-1	Methotrexate	Robust therapeutic effect in vivo	[296]

Programmed cell death protein 1 (PD-1), programmed death-ligand 1 (PD-L1), cytotoxic-T-lymphocyte-associated protein 4 (CTLA-4), interferon-beta (IFNβ), regulatory T cells (Tregs), C-X-C chemokine receptor type 10 (CXCR10), tumor microenvironment (TME), tumor-infiltrating lymphocytes (TILs), C–C chemokine receptor type 5 (CCR5), antigen-presenting cell (APC)

### Radiotherapy

Previously, the aptitude of ionizing radiation underlies cell death, and the inflammatory response has been indicated [214]. However, such attributes of radiation have enticed the evolving attention to arouse or improve antitumor immunity [214]. Researchers have observed unexpected out-of-the-field (abscopal) responses in patients receiving radiation therapy throughout immunotherapy [215]. Increasing evidence has delivered the proof of the theory that ICIs combination therapy with radiotherapy can convince more preferred anti-tumor response versus tumor cell primarily by triggering immunogenic cell death and resultant infiltration and activities of T cells within the TME [216, 217]. Radiotherapy may restore the antitumor influence of immune ICIs by eliciting endogenous danger signals and cytokines, augmenting the presentation of tumor-associated antigens (TAA) on APC along with inspiring T cell anti-tumor immunity [218, 219]. Correspondingly, in the HCC mice model, radiotherapy plus anti-PD-L1 promoted CD8+ T cell infiltration and activation, supported enhanced interferon IFN-γ production potential of TILs, and enticed a lessening trend in Tregs and exhausted T cells [220]. Another report exemplified that PD-L1 and IDO were stimulated on tumor epithelia of pancreatic ductal adenocarcinoma (PDAC) cells following radiotherapy, suggesting that radiation therapy may prime PDAC for PD-1 blockade therapy or IDO inhibitor treatments. Moreover, such combination treatment led to the more excellent systemic IFN-γ response and a local expression of immune-activation genes, including CD28 and ICOS, than monotherapy [221]. Furthermore, evaluation of the safety and efficacy of the radiotherapy plus ICI in 59 patients, mainly with RCC or melanoma treated with radiotherapy during or within eight weeks of ICI administration, signified this therapeutic modality's safety and modest efficacy [222]. Similarly, another study on 133 cancer patients revealed that a combination of focal palliative radiation and CTLA-4 and/or PD-1 inhibitors were well tolerated, with manageable irAEs [223]. In contrast, other reports indicating that radiotherapy plus ICI may result in reduced median OS, median PFS, and median time‑to‑treatment failure compared with monotherapy with ICIs have described the importance of the execution of further studies [224]. Now, several studies based on ICIs plus radiotherapy are being carried out on patients with cHL, HNSCC, NSCLC, SCC, RCC, melanoma, pancreatic cancer, breast cancer, and also bladder cancer (e.g., NCT0441944, NCT04892849, NCT04454489, NCT04793737, NCT03275597, NCT04454528, NCT02311361, and NCT03693014).

### Cancer vaccines

Therapeutic cancer vaccines facilitate tumor regression, elimination of minimal residual disease (MRD), and also establishing the long-term antitumor memory and avoiding non-specific or adverse reactions [225, 226]. To date, FDA has approved three vaccines, including Bacillus Calmette-Guérin (BCG) live, sipuleucel-T, and also talimogene laherparepvec (TVEC) for patients with early-stage bladder cancer, prostate cancer, and melanoma, respectively [227]. The FDA has approved the BCG Live (Intravesical) from 1990 in primary or recurrent UC upon transurethral resection [228]. Further, Sipuleucel-T gained approval from the FDA in April 2010 as autologous cellular immunotherapy used to treat metastatic castration-resistant prostate cancer (mCRPC) [229]. The FDA approved TVEC, a genetically modified oncolytic viral therapy in 2015 for advanced melanoma therapy [230]. Recently, shreds of preclinical evidence suggest that a combination of vaccines and ICIs may enhance immunogenicity and inhibit the immunosuppressive TME [231]. Cancer vaccines can bring about tumor-specific T cells in the periphery or situ tumors and ease infiltration of activated peripheral T cells into the TME [231]. Besides, vaccine-induced tumor cell elimination provokes the secretion of more cascade antigens and convinces more robust immune reaction specific to antigens not included within the vaccine, a phenomenon known as antigen cascade or epitope spreading [232]. So, the theory suggested that better ICI treatment efficacy may be attained by optimizing tumor immunogenicity or host immune reaction with vaccines [231].

In vivo, GM-CSF cell-based vaccines (GVAX) plus anti-CTLA-4 antibody has shown synergistic impacts in attenuating tumor size and restoring the antitumor immune reactions in melanoma [233] and also prostate [234] mice model. The DC tumor lysate-based vaccine plus PD-1 blockade also caused improved survival in glioma cell-bearing mice [235]. Besides, PD-1 blockade plus GVAX vaccine intensified survival and improved T-cell activity in mice with PDA [236]. On the other hand, a recent clinical trial (NCT01302496) showed that DC-based mRNA vaccination plus ipilimumab could lead to intense CD8+ T-cell responses in stage III or IV melanoma patients [237]. In addition, a study of the safety and efficacy of ipilimumab plus GVAX in 30 patients with advanced PDA previously treated showed that the intervention could, in some cases, improve the median OS compared to ipilimumab monotherapy [238]. Moreover, the efficacy of nivolumab was augmented by treatment with ISA 101, a synthetic long-peptide HPV-16 vaccine containing HPV-specific T cells, in patients with incurable HPV-16-positive tumors [239]. Based on the analysis, the overall response rate (ORR) of 33% and median OS of 17.5 months was more encouraging than monotherapy with nivolumab in similar patients [239].

Oncolytic viruses (OVs), as well-known types of cancer vaccines, are tumor-selective, multi-mechanistic antitumors [240, 241]. They lyse directly infected cancerous and endothelial cells while non-infected cells are destroyed by targeting cancer vessels and the bystander effect. Multimodal immunogenic cell death (ICD) accompanied with autophagy, which often is prompted by OVs, provides dominant danger signals to DCs and also supports cross-present tumor-associated antigens (TAAs) from tumor cells to DCs to T cells to trigger adaptive antitumor immunity [242]. Respecting the promising immune background, genetic engineering of OVs, and rational combinations efficiently can potentiate OVs as cancer vaccines [243, 244]. Notably, ICIs combination therapy with OVs has persuaded promising results in preclinical models primarily by stimulation of macrophage influx and M1-like polarization and stimulating recruitment and activities of T effector cells, improving IFN-γ levels in TME, and finally down-regulation of Treg and MDSCs density and activity [245, 246] (Table 6). Also, multiple ongoing clinical trials are currently combining OVs with ICIs (NCT02798406 and NCT03206073).

**Table 6:** Immune checkpoint inhibitors (ICIs) combination therapy with oncolytic viruses (OVs) (animal study)

Tumor	Target ICs	OVs type	Main result	References
Glioma	CTLA-4 PD-1	IL-12-expressing oHSV	Induction of macrophage influx and M1-like polarization and improving T effector (CD4+ and CD8+ T cells) to T regulatory cell ratio	[297, 298]
Rectal cancer	PD-1	hTERT-expressing oAd	Tumor regression by recruitment of CTLs	[299]
Osteosarcoma	PD-1	hTERT-expressing oAd	Tumor regression by recruitment of CTLs	[299]
Breast cancer	PD-1 CTLA-4	Soluble TGFβRIIFc-expressing oAd	Inhibition of tumor growth and lung and liver metastases	[300]
Lung cancer Breast cancer Melanoma Lymphoma	PD-1 PD-L1 CTLA-4	GM-CSF-expressing oHSV-1	Tumor regression and also induction of immunological memory	[300]
Glioblastoma multiforme (GBM)	PD-1	ZIKV	Improved survival of treated animals	[301]
Rhabdomyosarcoma	PD-1	oHSV	Amelioration of incidence of CD4+ and CD8+ T cells but not Treg populations in the tumor	[302]
Melanoma	PD-L1	oHSV	Enhancing IFNγ-producing CD8+ TILs Improved survival of treated animals	[303]
Lung adenocarcinoma	PD-1	oAd	Inhibition of tumor cell dissemination in a CD8 T-cell-dependent manner	[304]
Melanoma	PD-1 PD-L1 CTLA-4	CD40L-expressing oAd	Increasing the systemic level of tumor-specific CD8+ T cells, and also promoting the ratio of intratumoral CD8+ T cells to Treg	[245]
GBM	PD-L1	CD40L-expressing oAd	Inhibition of tumor development associated with increased survival	[305]
Prostate cancer	PD-1	oAd	Induction of antigen-specific CD8+ T-cell responses in mice	[306]
Melanoma	PD-1	oAd	Delayed tumor growth leading to the boosted survival of treated animal	[307]
Melanoma	PD-1	Reovirus	Enhanced capacity of NK cells to eliminate reovirus-infected tumor cells, abridged Treg activity and augmented the CD8+ T-cell-mediated antitumor response	[308]
GBM	PD-1	Reovirus	Improving the expression of IFN-regulated gene expression, as well as the PD-1/PD-L1 axis in tumors	[309]
GBM	PD-1 CTLA-4	HIF-2α, Sox-10, c-Myc, and TRP1-expressing VSV	Restoring the antitumor Th1 interferon-γ and Th17 T cell responses	[310]
Melanoma	PD-L1	MV	Inducing tumor remission	[311]
GBM	PD-1	EGFR-expressing MV	Improved inflammatory cell influx into the brains of treated mice Enhanced overall survival in treated animal	[312]

Programmed cell death protein 1 (PD-1), programmed death-ligand 1 (PD-L1), cytotoxic-T-lymphocyte-associated protein 4 (CTLA-4), interferon-gamma (IFNγ), regulatory T cells (Tregs), tumor-infiltrating lymphocytes (TILs), natural killer (NK) cell, cytotoxic T lymphocytes (CTLs), oncolytic herpes simplex virus (oHSV), oncolytic adenovirus (oAd), measles virus (MV), vesicular stomatitis virus (VSV), zika virus (ZIKV), human telomerase reverse transcriptase (hTERT), transforming growth factor-beta receptor 2 fused with Fc protein (TGFβRIIFc), granulocyte–macrophage colony-stimulating factor (GM-CSF), Hypoxia-inducible factor-2α (HIF-2α), SRY-related HMG-box 10 (SOX10), epidermal growth factor receptor (EGFR)

### Anti-angiogenic agents

Abnormal vasculature is one of the most renowned properties of solid tumors and contributes to tumor immune evasion [247]. This aberration arises from the enhancement in the expression of pro-angiogenic factors, chiefly contributed to the regulation of the function and migration of immune cells [247]. Anti-angiogenic compounds thereby can normalize blood vessels and transform the TME from immunosuppressive to immune-supportive by heightening the infiltration and stimulation of immune cell activities. Axitinib, bevacizumab, cabozantinib, everolimus, lenalidomide, lenvatinib mesylate, pazopanib, ramucirumab, regorafenib, sorafenib, sunitinib, thalidomide, vandetanib and Ziv-aflibercept are FDA-approved angiogenesis inhibitors [248]. Recently, Wang et al. supposed that anti-angiogenesis therapy may defeat the innate resistance to PD-1/PD-L1 blockade in vascular endothelial growth factor A (VEGF-A)-overexpressed mice tumor models [249]. The safety and efficacy of combination immunotherapies with ICIs and bevacizumab, a humanized monoclonal antibody binding to VEGF-A, has been evaluated in clinical trials to treat patients with NSCLC nasopharyngeal carcinoma and also metastatic CRC (NCT04997382, NCT04872582, and NCT04659382). As described, atezolizumab, in combination with bevacizumab, paclitaxel, and carboplatin, is the first-line FDA-approved treatment for patients with NSCLC [250]. Moreover, bevacizumab has exposed a synergistic effect with atezolizumab on the median OS of patients with RCC [251]. Also, evaluation of combined nivolumab and bevacizumab activity in 38 women with relapsed ovarian cancer during a phase 2 study indicated that 11 patients showed an objective response (OR) to nivolumab plus bevacizumab. In contrast, nine patients exhibited a grade 3 or higher treatment-associated unwanted event [252]. The efficacy of the intervention was more eminent in the platinum-sensitive setting, and thereby an alternative combinational strategy seems to be required in the platinum-resistant setting [252].

In addition to the chemotherapeutic agent, radiotherapy, OVs, other cancer vaccines, and anti-angiogenic agents, other therapies such as CXCR4 blockade [253, 254] have demonstrated great application capacity to combination with ICI, circumventing tumor resistance to ICIs.

## Conclusion and prospect

As described, ICI has been pronounced as a game changeling toll to treat even metastatic or chemoresistant malignancies with unfavorable prognoses. New inhibitory checkpoints and their target molecules are being studied to evolve the application and efficacy of current immune checkpoint inhibition therapy. Notwithstanding, such novel treatments are not effective sufficient to be utilized alone but can improve the activity of existing therapy. Albeit, this synergism may cause a boosted occurrence and severity of irAEs. The irAEs associated with ICI therapies underlies significant morbidity for patients and the considerable cost to the healthcare system, restricting their utility in the clinic.

Meanwhile, overactivation of the immune system may result in autoimmune-like side effects, thus finally perturbing organ functions and necessitating discontinuation of therapy, hospital admission, or management with systemic immunosuppressive compounds [29, 172]. Understanding the corresponding mechanism of these untoward events and how they can be distinguished from the antitumor impacts of ICI and recognising additional biomarkers that predict the incidence of irAEs will enable further trials to hinder their onset facilitate patient outcomes. Also, ‘on-target off-tumor’ impacts have recently been pronounced, thereby the possible influences of ICIs on healthy tissue remain disquiet. Concerning the mechanism of action, earlier clinical trials usually excluded patients with an underlying autoimmune disorder such as inflammatory bowel diseases (IBD) and arthritis. It has not yet been proven that the existence of these diseases can cause complete contraindication. Whether patients with chronic immunosuppression will respond to treatment has not yet been answered. In addition, various types of ICIs did not exhibit robust activity in ‘cold’ TMEs. Approaches to stimulate a shift to ‘hot’ TMEs may augment the efficacy and grow the use of these therapies. Though numerous clinical trials evidenced that elevating the dose has no statistically substantial impact on tumor response, more investigation into optimal ICI dosing approaches and progress of dose escalation plans may be justified. Comprehensive studies are urgently required to identify biomarkers that could aid select patients who may advantage the most while also ducking robust toxicities. Respecting current evidence, tumors exhibiting a high PDL-1 expression level and TMI, MSI, or dMMR have higher response rates to PD-1 blockade [255].

Also, the frequency of tumor-infiltrating lymphocytes (TILs) is suggested as a potent indicator of response to ICIs. In this context, PD-L1 protein expression on tumor or immune cells arose as the chief potential prognostic biomarker for sensitivity to ICIs. A growing body of evidence infers that higher baseline PD-L1 and/or PD-1 on peripheral blood T cells is a sign that increases the probability of successful treatment with PD-1 inhibitors. Patients with a higher quantity of PD-L1+ T cells typically show a more favored objective response to PD-1 inhibitor therapy. In comparison, patients with a lower portion of regulatory T cells at baseline mainly demonstrate more irAEs [256]. 9 FDA drug approvals related to a specific PD-L1 threshold and companion diagnostic in various cancers, such as bladder cancer, NSCLC, TNBC, cervical cancer, and gastric/gastroesophageal junction cancer [257]. Of course, combination therapies using ICIs and chemotherapy or other therapeutic modalities may further constrain the predictive value of PD-L1 expression. Hence, additional studies are needed to institute a consistent and dynamic predictive biomarkers scheme that may vary across tumor types and indications. The scientist must be cautious about applying the ICIs linked to PD/PD-L1 status in the suitable, FDA-approved setting.

## Data Availability

Not applicable.

## Abbreviations

ICIs: Immune checkpoint inhibitors
CTLA-4: Cytotoxic T-lymphocyte antigen-4
PD-1: Programmed cell death protein 1
PD-L1: Programmed death-ligand 1
TME: Tumor microenvironment
Tregs: Regulatory T cells
MDSCs: Myeloid-derived suppressor cells
TILs: Tumor-infiltrating lymphocytes
CTLs: Cytotoxic T lymphocytes
APCs: Antigen-presenting cells
DCs: Dendritic cells
IFNγ: Interferon-gamma
OS: Overall survival
irAEs: Immune-related adverse event
PFS: Progression-free survival
